# Targeting Golgi–STING Signaling to Reprogram Innate and Adaptive Immunity for the Treatment of Implant‐Associated Infections

**DOI:** 10.1002/advs.75623

**Published:** 2026-05-10

**Authors:** Shicheng Huo, Naifeng Zhu, Zhuocheng Lyu, Yifei Liu, Zhenjiang Xu, Chun Zhou, Yaochao Zhao, Changgui Shi

**Affiliations:** ^1^ Department of Orthopedic Surgery, The Spine Surgical Center Second Affiliated Hospital of Naval Medical University Shanghai China; ^2^ Department of Joint Replacement, Sports Medicine, and Trauma, Department of Orthopedics, Renji Hospital Shanghai Jiaotong University School of Medicine Shanghai China

**Keywords:** cGAS–STING pathway, Golgi pH, immunotherapy, implant‐associated infection, neutrophil‐hitchhiking nanoplatform

## Abstract

Implant‐associated infections (IAIs) remain a significant clinical challenge due to the persistence of biofilms and the immunosuppressive microenvironment. This study presents a neutrophil‐hitchhiking ultrasound (US)‐driven nanoplatform (CS‐BT@MZ@NEs) designed to target Golgi pH modulation, activate immune pathways, and enhance antibacterial efficacy. The nanoplatform leverages neutrophil migration to cross the bone marrow–blood barrier and precisely deliver to deep infection sites. At the infection site, CS‐BT@MZ@NEs disrupts bacterial biofilms via US‐driven effects and modulates Golgi pH to activate the STING pathway. Golgi acidification promotes STING oligomerization and TBK1 activation, driving the release of interferons and pro‐inflammatory cytokines, which enhance macrophage M1 polarization, dendritic cell maturation, and T‐cell activation. In vivo, CS‐BT@MZ@NEs significantly reduced bacterial burden, suppressed myeloid‐derived suppressor cell infiltration, and increased CD4^+^ and CD8^+^ T‐cell responses. Furthermore, the nanoplatform induced robust immune memory, characterized by expanded memory T‐cell pools and elevated IgG levels, effectively preventing reinfection. Importantly, CS‐BT@MZ@NEs showed no systemic toxicity. These findings highlight CS‐BT@MZ@NEs as a novel therapeutic platform that integrates targeted delivery, Golgi pH modulation, and immune memory activation, offering a promising solution for IAIs treatment and advancing the field of nano‐immunotherapy.

## Graphical abstract

This study presents an ultrasound‐responsive nanoplatform, CS‐BT@MZ@NEs, with a BaTiO_3_/Mn‐Zif‐8 core and a chondroitin sulfate coating for Golgi targeting. By leveraging neutrophil hitchhiking, it enables targeted delivery to infection sites. Under ultrasound stimulation, CS‐BT@MZ@NEs generates ROS and modulates Golgi pH to activate cGAS–STING signaling, thereby disrupting biofilms and enhancing antibacterial immunity.

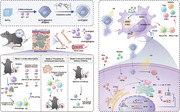

## 1 Introduction

Implant‐associated infections (IAIs) remain one of the most significant challenges in modern medicine, particularly in orthopedic, cardiovascular, and dental applications [1, 2, 3]. These infections arise due to the formation of bacterial biofilms, which consist of microbial communities encapsulated in extracellular polymeric substances (EPS) [4, 5]. Biofilms shield bacteria from both host immune responses and conventional antibiotics, rendering them highly resistant to treatment [6, 7]. Even after surgical debridement and implant replacement, residual biofilm fragments often lead to recurrent infections or chronic inflammation, severely impairing patient outcomes and increasing healthcare costs [8]. Traditional treatments for IAIs involve systemic antibiotics and surgical intervention, but their efficacy is limited. Antibiotics struggle to penetrate the dense biofilm structure, while the emergence of antibiotic‐resistant pathogens further exacerbates treatment challenges [9]. Moreover, biofilms create an immunosuppressive microenvironment, characterized by the infiltration of myeloid‐derived suppressor cells (MDSCs), reduced T‐cell activation, and impaired antigen presentation [10, 11, 12]. These factors compromise the host's immune defenses, making it difficult to clear infections and prevent reinfection.

To address these limitations, researchers have turned to advanced nanotechnology‐based strategies. Nanoparticles, in particular, offer a promising solution due to their ability to navigate complex biological environments, deliver therapeutic agents with precision, and modulate host immune responses [13, 14]. However, effective delivery of nanoparticles to deep‐seated infection sites, such as the bone marrow, remains a significant hurdle. The bone marrow–blood barrier and low blood perfusion pose challenges to achieving sufficient therapeutic concentrations at these sites [15, 16, 17]. To overcome these obstacles, leveraging the natural migratory properties of neutrophils offers a unique approach. Neutrophils, the most abundant white blood cells in the body, play a critical role in the innate immune response by migrating across biological barriers and homing to sites of infection or inflammation. By hitchhiking on neutrophils, nanoparticles can utilize these natural pathways to traverse the bone marrow–blood barrier and reach infection sites [18, 19, 20, 21]. This not only improves delivery efficiency but also capitalizes on neutrophils’ intrinsic antibacterial functions.

**SCHEME 1:**
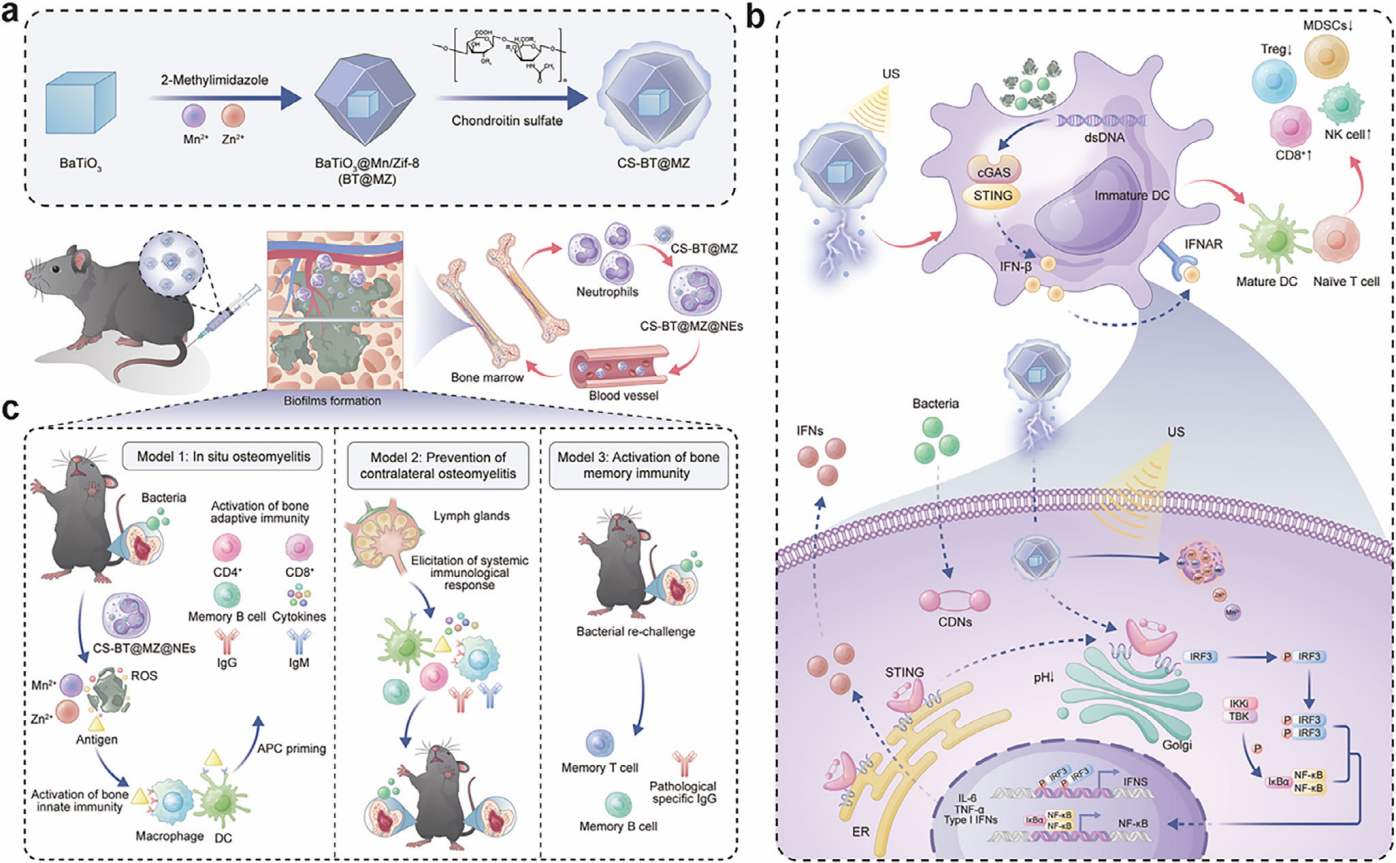
Schematic illustration of the construction, delivery, and therapeutic mechanism of CS‐BT@MZ@NEs for IAIs. (a) Preparation of CS‐BT@MZ and neutrophil‐mediated delivery to deep infection sites in bone marrow. (b) Under US stimulation, CS‐BT@MZ@NEs disrupts biofilms, promotes bacterial antigen exposure, and enhances Golgi acidification‐mediated STING activation, thereby stimulating innate and adaptive immune responses. (c) Therapeutic effects of CS‐BT@MZ@NEs in established infection, prevention of contralateral infection, and induction of immune memory against bacterial rechallenge.

In this study, we engineered an ultrasound (US)‐responsive nanoplatform, designated as CS‐BT@MZ. This nanoplatform features a core of barium titanate (BaTiO_3_, BT), renowned for its US piezoelectric properties, encapsulated within a manganese‐zinc metal‐organic framework (Mn‐Zif‐8, MZ), forming a functional heterostructure. Additionally, the nanoparticle surface is further coated with chondroitin sulfate (CS), known for its Golgi apparatus‐targeting capability, endowing the nanoplatform with targeting specificity and biocompatibility [22, 23, 24]. Subsequently, by co‐incubating with cultured neutrophils, we developed a CS‐BT@MZ@NEs‐loaded system, leveraging the innate migratory characteristics of neutrophils to achieve targeted delivery to infection sites. The Golgi apparatus plays a pivotal role in the activation of the cGAS‐STING signaling pathway, a crucial driver of innate immunity [25, 26]. An acidic Golgi environment is essential for STING oligomerization and subsequent recruitment of TBK1, which activates downstream immune signaling cascades (Scheme 1a). Dysregulation of Golgi pH can impair STING activation, reducing the host's ability to mount an effective immune response [27, 28].

CS‐BT@MZ@NEs addresses this challenge by restoring Golgi acidification. Upon delivery to the Golgi apparatus, the nanoplatform utilizes its US‐responsive properties to release H^+^ ions locally, reestablishing an optimal acidic environment. This mechanism enhances STING activation, leading to the robust production of interferons and pro‐inflammatory cytokines, such as TNF‐α and IL‐6. These cytokines drive macrophage M1 polarization, promote dendritic cells (DCs) maturation, and stimulate T‐cell activation, bridging innate and adaptive immunity (Scheme 1b). By targeting the Golgi apparatus, the nanoplatform amplifies immune responses with high specificity and efficacy. Furthermore, CS‐BT@MZ@NEs exhibits potent biofilm‐disrupting activity. Its US‐triggered mechanical effects break down the biofilm EPS matrix, exposing bacterial antigens and facilitating immune recognition, which helps overcome biofilm‐associated immune evasion. Additionally, the nanoplatform suppresses the infiltration of MDSCs, reducing the immunosuppressive effects of the IME and further enhancing the host's immune response. In vivo studies revealed that CS‐BT@MZ@NEs significantly reduced bacterial burden at infection sites, improved bone marrow immune responses, and prevented bacterial dissemination. Notably, the nanoplatform induced robust immune memory, as evidenced by increased populations of CD4^+^ and CD8^+^ memory T cells and elevated IgG levels in serum and bone marrow. This long‐lasting immune memory effectively protected against bacterial reinfection, underscoring the potential of CS‐BT@MZ@NEs as a prophylactic and therapeutic platform (Scheme 1c).

In conclusion, this study presents a multifunctional nanoplatform that integrates Golgi pH modulation, immune activation, and biofilm disruption to address the therapeutic challenges of IAIs. By leveraging neutrophil hitchhiking for targeted delivery and promoting STING activation through Golgi acidification, CS‐BT@MZ@NEs provides an integrated strategy for controlling biofilm‐associated infection and preventing recurrence. In the following sections, we describe the synthesis, characterization, and biological evaluation of CS‐BT@MZ@NEs and demonstrate its therapeutic potential in preclinical IAI models.

## 2 Results and Discussion

### 2.1 Synthesis and Characterization of CS‐BT@MZ Nanoplatform

**FIGURE 1:**
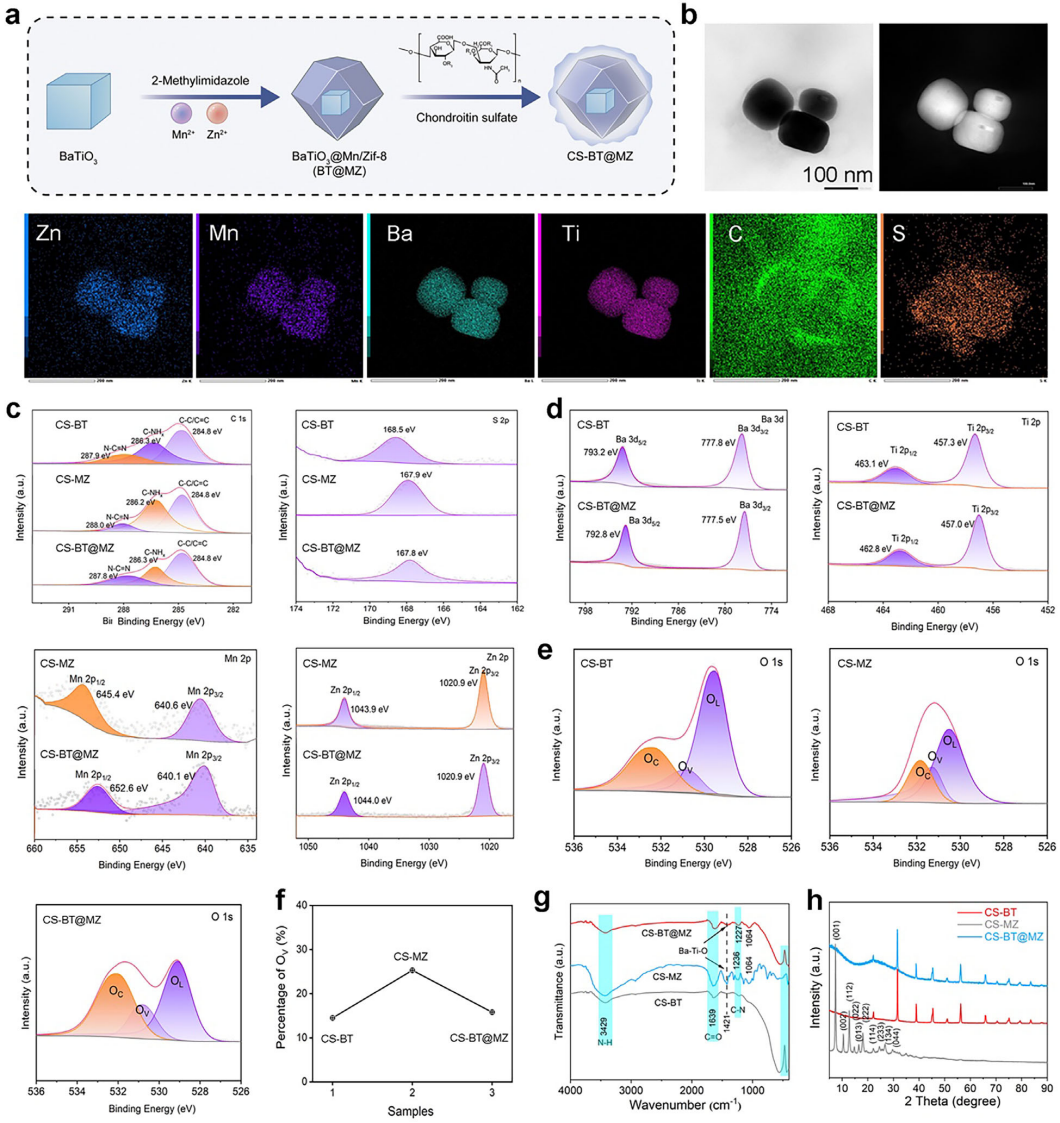
Schematics of CS‐BT@MZ construction and characterization. (a) Schematic illustration for the construction of CS‐BT@MZ. (b) Representative TEM and element‐mapping images of CS‐BT@MZ. (c,d) X‐ray photoelectron spectroscopy (XPS) spectra of C 1s, S 2p, Ba sd, Ti 2p, Mn 2p, and Zn 2p of various samples. (e,f) X‐ray photoelectron spectroscopy (XPS) spectra of O 1s of various samples and changes in the area ratio of OV/OL in these samples. (g) FTIR of various samples. (h) XRD of various samples.

Figure 1a illustrates the schematic synthesis of CS‐BT@MZ. The process encompasses several critical steps: initially, the core nanoparticles BT@MZ are synthesized, with a structure based on BT, modified with manganese (Mn) and zinc (Zn) ions to enhance functional properties. Subsequently, the nanoparticles are surface‐coated with CS, forming an outer functionalized coating layer. CS not only imparts excellent biocompatibility to the nanoparticles but also optimizes targeting capabilities through its specific affinity for the Golgi apparatus. The characterization of various nanocomposites was conducted using scanning electron microscopy (SEM), and the results revealed the successful synthesis of BT@MZ, exhibiting relatively uniform dimensions with an average particle size of approximately 100 nm (Figure S1). Dynamic light scattering (DLS) results further indicated an increase in particle size to 128.7 ± 0.55 nm following the coating with CS (Figure S2), confirming the successful encapsulation of CS. Additionally, transmission electron microscopy (TEM) images clearly displayed the CS coating layer, and elemental analysis maps demonstrated the presence of C and S elements within the coating (Figure 1b; Figure S3). Collectively, these findings validate the successful synthesis of CS‐BT@MZ, providing a robust structural foundation and characterization support for subsequent investigations.

X‐ray photoelectron spectroscopy (XPS) analysis indicates that during the coating process, the binding energies of C, Ti, Ba, Mn, and Zn elements remain largely unchanged (Figure 1c,d). This suggests that the structural integrity and chemical states of these elements are well‐preserved throughout the synthesis. The unchanged binding energies indicate that the functionalization process preserves the core properties of the material. Such stability is crucial for the material's performance within biological systems and its ability to respond to external stimuli, such as US. The oxygen vacancy content of various nanoparticles was analyzed using XPS. The results indicated that CS‐MZ exhibited the highest proportion of oxygen vacancies, while CS‐BT had the lowest, with CS‐BT@MZ falling in between (Figure 1e,f). This trend indicates that compositional modulation affects oxygen vacancy formation in the nanoparticles. Mn and Zn ions promote vacancy generation, whereas the BT structure exhibits higher lattice stability and fewer vacancies. Because excessive oxygen vacancies can disrupt charge distribution and weaken piezoelectric performance, the intermediate vacancy level in CS‐BT@MZ provides a balanced condition that preserves piezoelectric activity, supporting its US‐responsive functions.

Figure 1g illustrates the Fourier Transform Infrared (FTIR) spectral peaks of CS‐BT@MZ, elucidating the molecular structure and compositional details of the material. The minor peak at 1731 cm^−^^1^ corresponds to the stretching vibration of C═O, indicating the potential presence of carbonyl functional groups on the nanoparticle surface. Similarly, the peak at 1639 cm^−^^1^ is attributed to C═O stretching vibrations, further corroborating the existence of carbonyl groups. The characteristic peak at 1421 cm^−^^1^ is associated with the bending vibrations of Ba─Ti─O, signifying the presence of a barium titanate core. The doublet peaks at 1236 and 1227 cm^−^^1^ correspond to C─N stretching vibrations, possibly originating from amino modifications or organic molecular components on the nanoparticle surface. The characteristic peak at 422 cm^−^^1^ is attributed to Ti─O vibrations, further confirming the structural integrity of barium titanate [29]. Figure 1h illustrates the X‐ray diffraction (XRD) patterns of various nanocomposites, clearly delineating the crystalline structural characteristics of each material. For CS‐BT, the XRD pattern exhibits a prototypical perovskite‐type crystal structure, with characteristic diffraction peaks at 2θ = 22.8°, 31.7°, and 45.5°, corresponding to the (100), (110), and (200) planes of BT, respectively. These distinctive peaks indicate that the core structure of barium titanate remains intact during functionalization. The pattern for CS‐MZ reveals markedly different diffraction features, with the distribution of characteristic peaks reflecting the impact of manganese‐zinc (Mn‐Zn) ion modification on the material's crystal structure. This structural formation may endow the material with unique functional properties, such as enhanced chemical reactivity or improved surface characteristics. In the XRD pattern of CS‐BT@MZ, diffraction features of both CS‐BT and CS‐MZ are observed, albeit with slight variations in overall peak intensity. This variation suggests the successful synthesis of CS‐BT@MZ, with limited impact of surface modification on the core crystal structure, primarily affecting surface properties. Additionally, the encapsulated CS does not exhibit distinct diffraction peaks, consistent with its amorphous nature.

Using inductively coupled plasma mass spectrometry (ICP‐MS), the concentrations of Mn^2^^+^ and Zn^2^^+^ in CS‐BT@MZ were determined to be 2.78 and 7.64 wt%, respectively (Figure S4a). The influence of US on the release of Mn^2^^+^ and Zn^2^^+^ from CS‐BT@MZ was further investigated. The findings indicate that CS‐BT@MZ exhibits remarkable responsiveness under US stimulation, significantly enhancing the release efficiency of metal ions. Under US, approximately 77.3% of Mn^2^^+^ was released from CS‐BT@MZ within 24 h (Figure S4b), which is substantially higher than the 44.6% observed without US. Similarly, the release proportion of Zn^2+^ reached 65.8% under US, compared to 38.13% without US (Figure S4c). In addition to supporting the US‐responsive physicochemical activity of the nanoplatform, the released metal ions may also provide complementary biological effects. Zn^2^^+^ has been reported to interfere with bacterial membrane integrity and metabolic processes, thereby contributing to antibacterial activity. Meanwhile, Mn^2^^+^ has been implicated in immune‐related signaling and may participate in the regulation of host immune responses. Therefore, the release of Mn^2^^+^ and Zn^2^^+^ may act synergistically with the piezoelectric ROS generation to enhance both antibacterial and immunomodulatory effects of the system.

**FIGURE 2:**
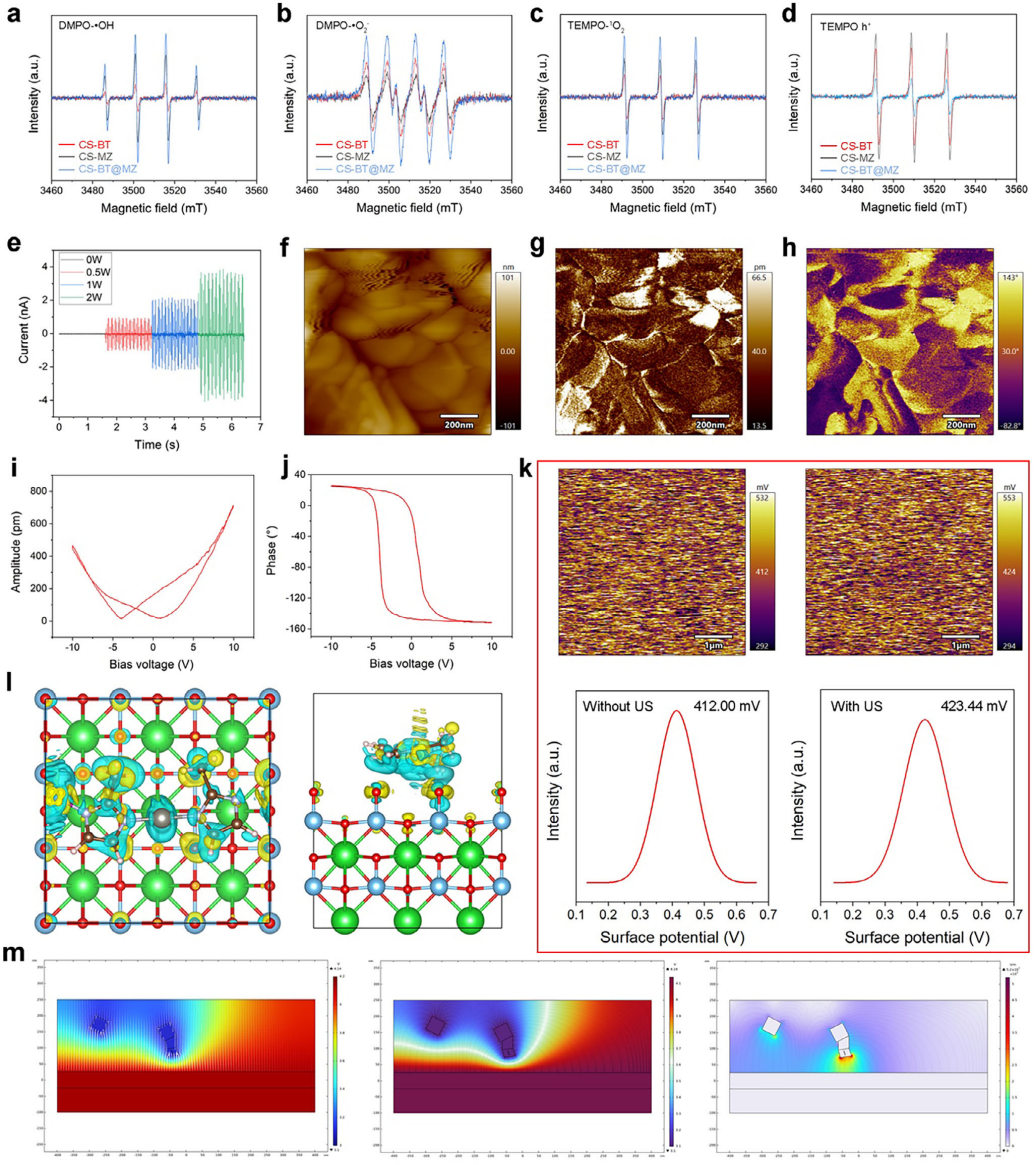
Characterization of CS‐BT@MZ nanoplatform. (a–d) ESR of CS‐BT@MZ. (e) Output current of CS‐BT@MZ under US stimulation. (f) PFM image of the surface of CS‐BT@MZ. (g) Piezoelectric amplitude of CS‐BT@MZ. (h) Phase angle of CS‐BT@MZ. (i) Butterfly cycle curve of CS‐BT@MZ. (j) Piezoelectric flip curve of CS‐BT@MZ. (k) KPFM images and corresponding surface potential distributions (With or without US) of CS‐BT@MZ. (l) Charge density distribution of BT/Mn‐Zif heterostructure. (m) Multiphysics field modeling to simulate the electric fled of BT/Mn‐Zif heterostructure.

Further investigation was conducted on the capability of the nanoplatform CS‐BT@MZ to generate ROS under US stimulation, including hydroxyl radicals (•OH), singlet oxygen (^1^O_2_), and superoxide radicals (•O_2_^−^), while also monitoring the production of holes (h^+^). As depicted in Figure 2a–d, the ESR spectra clearly exhibited characteristic peaks for •OH, •O_2_^−^, ^1^O_2_, and h^+^ under US conditions, indicating that the nanoplatform efficiently generates multiple ROS when subjected to US. Notably, the peak intensities of •OH, •O_2_^−^, and ^1^O_2_ for CS‐BT@MZ were significantly higher than those of other control groups, suggesting its superior ROS generation capability under US stimulation. This enhanced ROS production is likely attributed to the material design of CS‐BT@MZ, which effectively facilitates electron transfer and ROS generation through the piezoelectric effect induced by US. Moreover, for holes (h^+^), CS‐BT@MZ exhibited the lowest peak intensity, indicating a material design that favors efficient separation of electrons and holes, thereby preventing electron‐hole recombination and further enhancing ROS generation efficiency. To further validate the piezoelectric conversion capability of CS‐BT@MZ, we examined its output voltage response under US pressure. As depicted in Figure 2e, CS‐BT@MZ exhibited a pronounced output current signal when subjected to dynamic US pressure, with the signal intensity significantly increasing alongside the US power. This linear relationship indicates that CS‐BT@MZ can efficiently convert the mechanical energy of US into electrical signals, highlighting its exceptional piezoelectric properties. PFM analysis was performed to evaluate the local piezoelectric response of CS‐BT@MZ. Under a 10 V tip bias, CS‐BT@MZ exhibited a piezoelectric amplitude of 66.5 pm and a phase angle of 143° (Figure 2g,h), indicating a pronounced surface piezoelectric response. In addition, the characteristic butterfly amplitude loops and phase hysteresis loops (Figure 2i,j) revealed non‐zero remanent polarization and typical ferroelectric behavior, confirming the strong piezoelectric and polarization properties of CS‐BT@MZ. To investigate the spatial distribution of acoustically induced charge transfer in CS‐BT@MZ, in situ US irradiation Kelvin probe force microscopy (KPFM) was employed. As depicted in Figure 2k, under US irradiation, the average surface potential of CS‐BT@MZ significantly increased to 423.44 mV, compared to 412 mV in the absence of US, indicating a difference of 11.44 mV. This result demonstrates that US irradiation effectively stimulates the charge transfer process in CS‐BT@MZ, further corroborating the piezoelectric effect and charge separation capability of the nanoplatform under US stimulation. These results further validate the piezoelectric performance and ferroelectric properties of CS‐BT@MZ, establishing a solid foundation for its multifunctional applications in US‐driven environments. The efficient piezoelectric response not only supports stable operation in US environments but also provides a reliable energy foundation for ROS generation, local acidic environment regulation, and precise modulation of Golgi apparatus functions.

To further elucidate the charge transfer mechanism of CS‐BT@MZ, we simulated its charge density distribution (Figure 2l). The results indicate that electrons are transferred from BT to Mn‐Zif through the heterostructure, confirming that this architecture facilitates carrier separation and transfer. This efficient charge transfer behavior provides a theoretical basis for the enhanced sonodynamic effect of the nanoplatform under US irradiation. Furthermore, COMSOL Multiphysics simulation was used to analyze the spatial electric potential distribution and electric field direction of the CS‐BT@MZ piezoelectric heterojunction (Figure 2m). The results show that the built‐in electric field at the BT/Mn‐Zif interface is directed from BT to Mn‐Zif, which favors the separation of electron–hole pairs generated under US stimulation and suppresses charge recombination. Together, these results suggest that the heterostructure of CS‐BT@MZ enhances sonodynamic activity and ROS generation by promoting charge transfer and carrier separation.

### 2.2 Cytocompatibility Evaluation and Optimization of Antibacterial Working Conditions

The in vitro cytocompatibility of CS‐BT@MZ was evaluated using rBMSCs. As shown in Figure S5a,b, CS‐BT@MZ maintained high cell viability at concentrations up to 100 µg/mL, whereas obvious cytotoxicity was observed at higher concentrations. IC50 analysis further indicated that US treatment moderately affected the cytotoxic profile of the material, as reflected by the difference between the US (+) and US (−) groups. Based on these results, 100 µg/mL was selected for subsequent cytocompatibility evaluation. Live/dead staining further showed predominantly viable cells with negligible dead‐cell signals in all treatment groups at the working concentration (Figure S5c). Consistently, cytoskeletal staining demonstrated that the cells retained normal spreading and morphology without obvious structural damage (Figure S5d). These results indicate that CS‐BT@MZ exhibits acceptable cytocompatibility toward mammalian cells under the experimental conditions used in this study.

On this basis, the antibacterial concentration of CS‐BT@MZ was optimized by in vitro plate‐counting assays under a fixed US condition of 1.5 W cm^−^^2^ for 5 min. Different concentrations of CS‐BT@MZ (0, 10, 50, 100, 200, and 500 µg mL^−^^1^) were evaluated. As shown in Figure S6a, the antibacterial efficacy gradually increased with increasing concentration, and a marked inhibitory effect was observed when the concentration reached 100 µg/mL or above. Considering both antibacterial performance and cytocompatibility, 100 µg/mL was selected as the working concentration for subsequent experiments.

The effect of US intensity on antibacterial performance was then examined under a fixed treatment time of 5 min. Different US power densities (0, 0.5, 1.0, 1.5, 2.0, and 2.5 W/cm^2^) were applied. The results showed that the antibacterial effect of CS‐BT@MZ was further enhanced with increasing US intensity, with a significant improvement observed at 1.5 W/cm^2^ and above (Figure S6b,c). Therefore, 1.5 W/cm^2^ was selected as the US condition for the following experiments.

### 2.3 CS‐BT@MZ+US Modulates Biofilm Antigen Expression

The formation of biofilms is a critical factor in bacterial evasion of host immune defenses, further complicating the treatment of IAIs using conventional methods. Research indicates that biofilms formed during infection not only serve as protective barriers against immune cells but also function as predatory mechanisms that effectively eliminate immune cells [30, 31]. Bacterial biofilms have evolved various strategies to evade immune system detection, including mimicking host cell structures, modulating antigen expression, and secreting EPS [32]. These mechanisms not only provide a physical barrier for bacteria but also impair immune signaling and effector function. In addition, the structural complexity of biofilms limits immune cell infiltration and activity, allowing bacteria to persist over time. Therefore, promoting biofilm antigen exposure has become a key strategy for enhancing immune recognition and activating innate and adaptive immune responses against IAIs.

**FIGURE 3:**
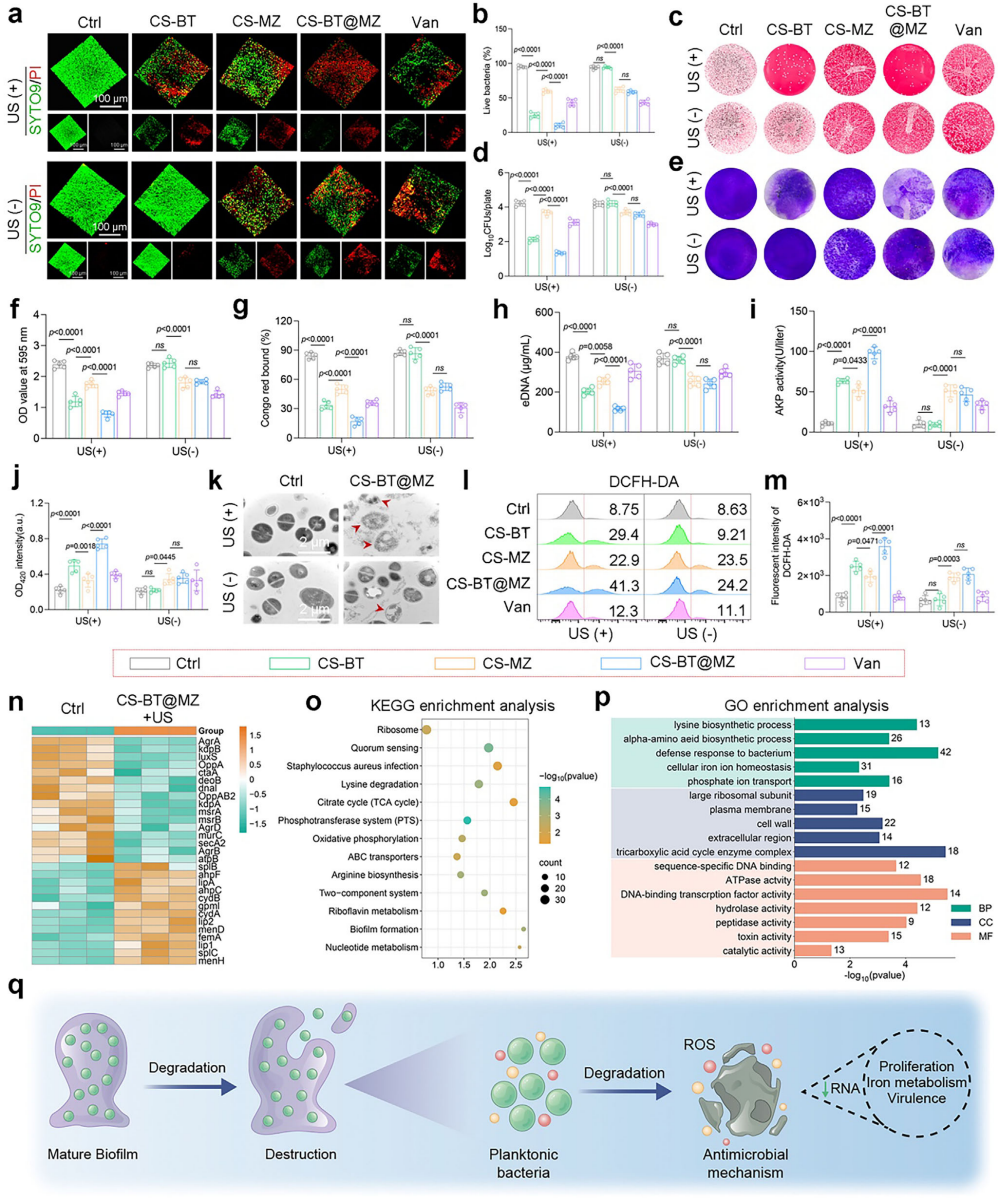
Anti‐biofilm activity of CS‐BT@MZ + US. (a) Live/dead staining of biofilms after different treatments. (b) Disruption of biofilms and embedded bacteria. (c,d) SPM images and quantitative analysis of bacteria in biofilms. (e) Crystal violet staining of biofilms after different treatments. (f) Quantification of crystal violet absorbance at 595 nm. (g,h) Quantification of exopolysaccharides and eDNA after treatments. (i,j) Alkaline phosphatase (AKP) activity and ONPG hydrolysis assay of treated bacteria. (k) TEM images showing bacterial morphology in biofilms after treatments. (l,m) ROS generation detected by DCFH‐DA using flow cytometry and microplate reader. (n) Heatmap of differentially expressed genes in the CS‐BT@MZ + US group. (o) KEGG pathway enrichment. (p) GO enrichment. (q) Schematic illustration of CS‐BT@MZ + US‐induced biofilm antigen exposure. Data are presented as mean ± SD (n = 5 per group), with “n” denoting biologically independent experiments.

As illustrated in Figure 3a,b, the CS‐BT@MZ + US treatment significantly disrupted the architecture of mature biofilms and effectively reduced the number of viable bacteria within them. This indicates that the acoustically driven nanoplatform can effectively compromise the integrity of the biofilm matrix through mechanical forces and chemical reactivity, markedly diminishing its function as a protective barrier for bacteria. The study highlights the pivotal role of EPS degradation in releasing bacterial antigens and enhancing immune cell recognition. The degradation of EPS not only destabilizes the biofilm but also exposes concealed bacterial antigens, thereby augmenting the host immune system's ability to detect pathogens. Further quantitative analysis (Figure 3c,d) demonstrated a significant reduction in the number of viable bacteria within the biofilms in the CS‐BT@MZ + US treatment group compared to other groups, indicating superior antibacterial efficacy. Additionally, biofilms were stained using a 1% crystal violet staining method, and the absorbance of treated samples was measured with a spectrophotometer after 24 h of incubation at 37°C (Figure 3e,f). The results showed that the CS‐BT@MZ + US treatment significantly reduced bacterial adhesion to implant surfaces and substantially weakened the structural integrity of the biofilms. Moreover, as depicted in Figure 3g,h, the CS‐BT@MZ + US treatment demonstrated a pronounced capability to disrupt key components of the biofilm matrix, including polysaccharides and extracellular DNA (eDNA). These matrix constituents are crucial for maintaining the structural integrity and stability of biofilms, and their disruption is pivotal for inhibiting biofilm formation. Among all evaluated treatments, CS‐BT@MZ + US exhibited the most significant inhibitory effect on polysaccharides and eDNA. Specifically, compared to the control group cultured with CS‐BT@MZ alone, the CS‐BT@MZ + US treatment significantly reduced the eDNA biomass to 122.3 ± 2.5 µg/mL after 5 min of US application, indicating that US‐assisted nanoplatform can effectively degrade the core components of the biofilm matrix.

After staining the bacteria from each treatment group with live/dead dyes, their viability was assessed using fluorescence microscopy (Figure S6d). The results indicated that the CS‐BT@MZ + US groups exhibited significant cell death, as evidenced by intense red staining signals. This suggests that the CS‐BT@MZ + US group possesses higher antibacterial efficacy compared to groups with fewer dead cells (Figure S6e). These findings were further corroborated by SPM (Figure S6f). Additionally, flow cytometry (FCM) analysis revealed a marked increase in bacterial apoptosis following CS‐BT@MZ + US treatment, as indicated by a higher propidium iodide (PI) positivity rate (Figure S6g). To quantitatively validate this phenomenon, the fluorescence intensity of PI was measured using a microplate reader. The results further confirmed that the PI fluorescence intensity in the CS‐BT@MZ + US treatment group was significantly higher than in other treatment groups (Figure S6h).

The mechanism by which CS‐BT@MZ + US treatment affects *MRSA* embedded in mature biofilms was further investigated by assessing extracellular alkaline phosphatase (AKP) levels and conducting the 2‐nitrophenyl‐beta‐d‐galactopyranoside (ONPG) hydrolysis assay. Results indicated a significant increase in extracellular AKP concentration following CS‐BT@MZ + US treatment (Figure 3i), with AKP release serving as a critical indicator of bacterial cell wall damage. This suggests substantial disruption of the bacterial cell wall by the treatment. The ONPG hydrolysis assay further demonstrated that ONPG molecules could penetrate the bacterial cytoplasm and be cleaved by intracellular β‐galactosidase to produce o‐nitrophenol, indicating a marked increase in bacterial cell membrane permeability. Notably, the absorbance value measured at 420 nm significantly increased post‐treatment (Figure 3j), corroborating the enhanced permeability of the bacterial cell membrane. Additionally, TEM analysis revealed profound effects of CS‐BT@MZ + US treatment on bacterial structure. Treated bacteria exhibited pronounced cell membrane contraction, blurred boundaries, and a corrugated morphology, whereas the control PBS group bacteria displayed smooth, intact, and undamaged cell membranes (Figure 3k). These observations suggest that CS‐BT@MZ + US treatment effectively eradicates bacteria embedded in biofilms by compromising the integrity of bacterial cell walls and membranes, thereby facilitating further immune system responses. Importantly, the remnants of apoptotic and damaged bacteria serve as significant sources of bacterial antigens. Studies indicate that these antigens can be efficiently internalized by DCs, promoting cross‐presentation of antigens and stimulating downstream adaptive immune responses [33, 34]. These findings underscore the potential of CS‐BT@MZ + US in disrupting biofilms, enhancing immune recognition, and bactericidal activity, offering novel insights into immuno‐combinatorial therapies for IAIs.

Subsequently, we delved deeper into the bactericidal mechanisms mediated by GMBC + US. In our previous investigations, we confirmed that the CS‐BT@MZ + US treatment generates a substantial amount of ROS (Figure 2a–d), suggesting that its antimicrobial properties might be attributed to ROS activity. As illustrated in Figure 3l,m, there is a marked increase in ROS production within bacteria following CS‐BT@MZ + US treatment. Further analysis indicates that this therapeutic approach likely employs the synergistic action of ROS to penetrate the biofilm surface layer by layer, effectively compromising the integrity of bacterial cell membranes and thereby achieving potent biofilm disruption. Notably, beyond the EPS barrier of biofilms, the persistent presence of viable bacteria within the biofilm poses another significant challenge in treating *MRSA*. The findings demonstrate that CS‐BT@MZ + US not only effectively penetrates *MRSA* biofilms but also generates multiple synergistic active substances under the influence of US. These active substances not only enhance the antibacterial efficacy of ROS but also further facilitate biofilm degradation and bacterial eradication.

To further explore the antibacterial mechanism of CS‐BT@MZ + US against MRSA biofilms, we performed RNA sequencing on the CS‐BT@MZ + US group and the control group, each with three replicates. CS‐BT@MZ + US markedly reshaped the bacterial transcriptional profile, with 642 genes downregulated and 669 genes upregulated among 2622 expressed genes (Figure S7a). The volcano plot further illustrates the distribution of these differentially expressed genes, while correlation analysis confirmed good comparability between the two groups (Figure S7b). Differentially expressed genes (DEGs) heatmap analysis (Figure 3n) reveals significant downregulation of genes associated with quorum sensing (QS) systems and bacterial biofilm formation, such as AgrA, AgrB, AgrD, and secA2, in the CS‐BT@MZ + US treatment group. Additionally, genes related to cell membrane integrity, like kdpA and kdpB, also exhibited downregulated expression in the CS‐BT@MZ + US group. Notably, the CS‐BT@MZ + US treatment significantly impacted bacterial manganese ion homeostasis. Specifically, the manganese ion uptake gene (mend and menH) was upregulated, while the manganese ion efflux gene (deoB) was downregulated. This may be attributed to the synergistic effects of sonodynamic therapy (SDT) and chemodynamic therapy (CDT), where CS‐BT@MZ + US generates a substantial amount of ROS, compromising bacterial cell wall and membrane integrity and affecting proton transport channels, thereby increasing the influx of metal ions into the bacterial cytoplasm. KEGG analysis further revealed significant disruption of pathways related to ribosomal function and bacterial metabolism (Figure 3o). This effect may be attributed to ROS‐mediated membrane damage, which increases membrane permeability and impairs intracellular biosynthetic processes, thereby disrupting ribosomal function. Simultaneously, Gene Ontology (GO) enrichment analysis results further corroborated these observations (Figure 3p). The results showed significant impacts on molecular functions such as ribosomal subunit, rRNA binding, protein‐containing complex, ribonucleoprotein complex, and structural molecular activity, indicating severe disruption of bacterial metabolic and biosynthetic processes. These changes are closely associated with a marked decline in bacterial survival capability.

In summary, CS‐BT@MZ + US eradicates bacteria by disrupting bacterial biofilms, utilizing ROS‐related oxidative stress toxicity to induce bacterial DNA damage and protein leakage, inhibiting the tricarboxylic acid (TCA) cycle (Figure 3q). These findings provide crucial experimental evidence for further development of ROS‐based anti‐biofilm therapeutic strategies and highlight the potential application value of CS‐BT@MZ + US as an anti‐infective therapy.

### 2.4 Golgi Targeting and pH Modulation of CS‐BT@MZ

**FIGURE 4:**
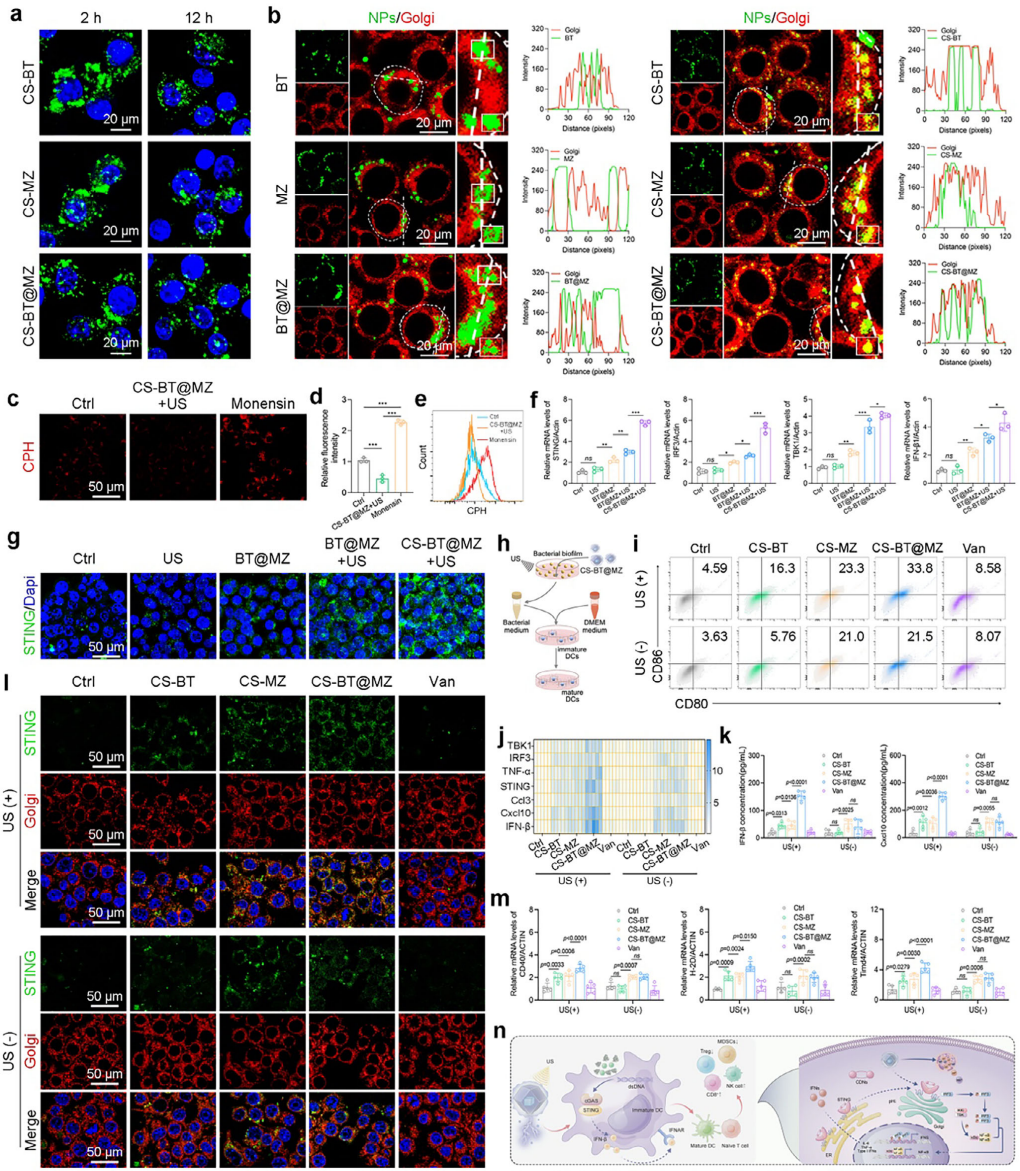
In vitro Golgi targeting, pH modulation, and DC maturation induced by CS‐BT@MZ. (a) Confocal images showing uptake of different NPs in BMDMs at 2 and 12 h. (b) Co‐localization of nanoparticles with the Golgi apparatus in BMDMs. (c) Fluorescence imaging of Golgi pH probes after different treatment. (d) Quantification of normalized fluorescence intensity. (e) Flow cytometry analysis of Golgi pH probe fluorescence after treatments. (f) Expression of STING‐related genes. (g) STING protein expression in BMDMs after different treatments. (h) Preparation of biofilm‐conditioned medium (BCM) and DC‐conditioned medium (DCM). (i) Quantification of mature DCs (CD80^+^CD86^+^) by flow cytometry. (j) Relative expression of immune activation‐related genes in DCs treated with BCMs. (k) Secretion of IFN‐β and CXCL10 by DCs after BCM treatment. (l) Immunofluorescence images showing STING expression and its Golgi‐associated localization under different BCM stimulation conditions. (m) Expression of antigen presentation‐related genes in DCs after BCM treatment. (n) Schematic illustration of CS‐BT@MZ‐mediated Golgi pH modulation and STING activation promoting DC maturation. Data are presented as mean ± SD (n = 5 per group), with “n” denoting biologically independent experiments.

To ascertain the ability of various NPs to effectively penetrate cellular interiors, we evaluated their phagocytic efficiency in BMDMs. The findings revealed that these NPs exhibit a uniform size distribution and excellent stability. Moreover, the fluorescently labeled NPs (FITC‐NPs) demonstrated rapid and efficient internalization by BMDMs, as evidenced in Figure 4a, rather than merely adhering to the cell surface. These results indicate a robust intracellular entry capability of NPs, laying the groundwork for further functional studies. To further validate whether nanoparticles coated with CS can target the Golgi apparatus, we conducted colocalization experiments. As illustrated in Figure 4b, after co‐culturing FITC‐labeled nanoparticles (FITC‐NPs) with BMDMs for 2 h, the fluorescence signal (green) of nanoparticles without CS coating was distinctly separated from the Golgi‐selective dye (red), indicating these particles failed to localize to the Golgi apparatus. In contrast, FITC‐labeled CS‐coated nanoparticles (CS‐NPs) exhibited a pronounced Golgi localization signal, suggesting that CS‐NPs were effectively transported to the Golgi apparatus following endocytosis. The Golgi apparatus is rich in glycosyltransferases (GTs), glycosidases, and nucleotide sugars, which synergistically contribute to carbohydrate synthesis and covalently attach glycans to proteins [35, 36, 37]. Among these, N‐acetylgalactosaminyltransferases (GalNAc‐Ts) specifically recognize polypeptide chains and transfer N‐acetylgalactosamine (GalNAc) onto them [38]. Since GalNAc is a major component of CS, the affinity of CS‐NPs for the Golgi apparatus can be attributed to the specific interaction between GalNAc‐Ts and CS [39, 40]. This finding further corroborates the Golgi‐targeting capability of CS‐NPs, providing a theoretical basis for their application in modulating Golgi functions.

Subsequently, we employed the Golgi apparatus pH fluorescent probe CPH to investigate the effects of various treatments on the Golgi pH. Monensin was used as a positive control (as it induces oxidative stress in the Golgi, significantly increasing its pH), while PBS served as a negative control. BMDMs were pre‐treated with PBS, CS‐BT@MZ, and monensin for 7 h, followed by the addition of the fluorescent probe CPH. After a 25‐min incubation, confocal microscopy imaging was performed. Fluorescence emission wavelengths of 700–760 nm were collected under 633 nm laser excitation for analysis (Figure 4c,d). The results indicated that the fluorescence intensity of the probe in the monensin‐treated group was significantly enhanced (approximately 2.3 times that of the control group), suggesting that monensin treatment markedly increased the Golgi pH. In contrast, the fluorescence intensity in the CS‐BT@MZ + US treatment group was significantly lower than that of the PBS control group, indicating that CS‐BT@MZ + US effectively reduced the Golgi pH. These findings confirmed the role of CS‐BT@MZ + US in modulating Golgi acidity. In further experiments, cells treated and incubated with the probe were digested with trypsin, rinsed with PBS, and collected in 1.5 mL EP tubes for flow cytometry analysis. The experimental results were consistent with the CLSM findings, further demonstrating that CS‐BT@MZ + US treatment significantly reduced the Golgi pH (Figure 4e).

The reduction of Golgi pH by CS‐BT@MZ + US likely results from several coordinated processes. After endocytosis, the CS coating facilitates Golgi targeting, enabling the nanoparticles to accumulate in the Golgi region. Under US stimulation, the piezoelectric activity of CS‐BT@MZ generates localized electrochemical effects and ROS, which may disturb Golgi ion homeostasis and promote proton accumulation, thereby enhancing Golgi acidification. In addition, these effects may influence proton transport‐related processes on the Golgi membrane, further contributing to pH reduction. Together, these results suggest that CS‐BT@MZ + US reduces Golgi pH through the combined effects of Golgi targeting, US‐responsive piezoelectric activity, ROS generation, and local ion regulation, thereby facilitating downstream immune signaling and antibacterial responses.

Within the Golgi apparatus, the molecular structure of STING undergoes conformational changes, resulting in the formation of functional oligomeric complexes that are crucial for downstream signaling, such as the recruitment and phosphorylation of TBK1. Studies have demonstrated that the acidic pH environment of the Golgi apparatus is vital for maintaining the conformation and function of the STING protein. An optimal acidic environment not only facilitates STING oligomerization but also significantly enhances the recruitment efficiency of TBK1 kinase, thereby amplifying the signal transduction effect. Notably, CS‐BT@MZ + US, through its US‐driven mechanism, effectively regulates the pH of the Golgi apparatus, promoting the formation of an acidic environment. To further investigate the specific impact of the Golgi apparatus's acidic environment induced by CS‐BT@MZ + US on STING activation, we conducted subsequent validation experiments. Initially, we employed PCR technology to assess the impact of CS‐BT@MZ + US treatment on the expression of genes associated with the STING pathway (Figure 4f). The results indicated that, compared to the control group, CS‐BT@MZ + US significantly upregulated the expression levels of STING, IRF3, TBK1, and IFN‐β, suggesting that this treatment can activate key components of the STING signaling pathway. Furthermore, immunofluorescence analysis corroborated these findings, demonstrating a marked enhancement in the overall STING expression level in the CS‐BT@MZ + US treatment group (Figure 4g). These discoveries suggest that CS‐BT@MZ + US facilitates the activation of the STING signaling pathway by modulating Golgi pH, thereby providing a molecular basis for the enhancement of the host immune response.

Subsequently, we extracted the supernatant from MRSA biofilms subjected to various treatments and prepared it into biofilm‐conditioned media (BCMs) for culturing BMDCs (Figure 4h). The results indicated that the proportion of mature DCs (CD80^+^CD86^+^) significantly increased from 4.59% to 33.8% following CS‐BT@MZ + US treatment (Figure 4i). The maturation of DCs further led to a marked elevation in the expression levels of inflammatory cytokines and chemokines (Figure 4j,k), thereby enhancing the intensity of the innate immune response. Immunofluorescence analysis with Golgi co‐staining further showed enhanced Golgi‐associated STING activation under different BCM stimulation conditions, providing additional support for the role of Golgi regulation in STING signaling (Figure 4l). Moreover, in the CS‐BT@MZ + US group, the activation of the cGAS‐STING pathway resulted in a significant upregulation of gene expression related to antigen presentation and T cell migration within DCs (Figure 4m).

In summary, CS‐BT@MZ + US effectively activated the cGAS‐STING signaling pathway by significantly enhancing the release of bacterial antigens and promoting Golgi acidification. Furthermore, this treatment markedly facilitated the maturation of DCs, thereby augmenting the activation and efficacy of adaptive immunity (Figure 4n). Ultimately, this dual‐action mechanism synergistically enhanced the host's overall immune defense capabilities, offering new strategies and possibilities for the immunotherapy of biofilm infections.

### 2.5 In Vivo Distribution and Bone Marrow‐Targeting Tendency of CS‐BT@MZ@NEs

Low blood perfusion and the bone marrow–blood barrier severely restrict drug penetration into deep marrow tissue, creating a major obstacle for the treatment of osteomyelitis, IAIs, and other bone‐related diseases. Although a range of nanocarriers and targeting ligands have been developed to improve bone marrow delivery, their efficiency remains limited by poor vascular penetration and restricted access to the marrow niche. These limitations highlight the need for more effective delivery strategies for deep bone marrow targeting. Neutrophils possess unique migratory and lifecycle characteristics that make them attractive candidates for drug delivery [41, 42]. In particular, they are capable of crossing the bone marrow–blood barrier and homing to the bone marrow [43]. Previous studies have shown that neutrophils cultured in vitro for 6 h upregulate CXCR4 expression, which promotes their return to the bone marrow through interaction with CXCL12 (SDF‐1) in the marrow microenvironment [44]. These properties make neutrophils a promising carrier for delivering therapeutic agents to deep bone marrow sites.

**FIGURE 5:**
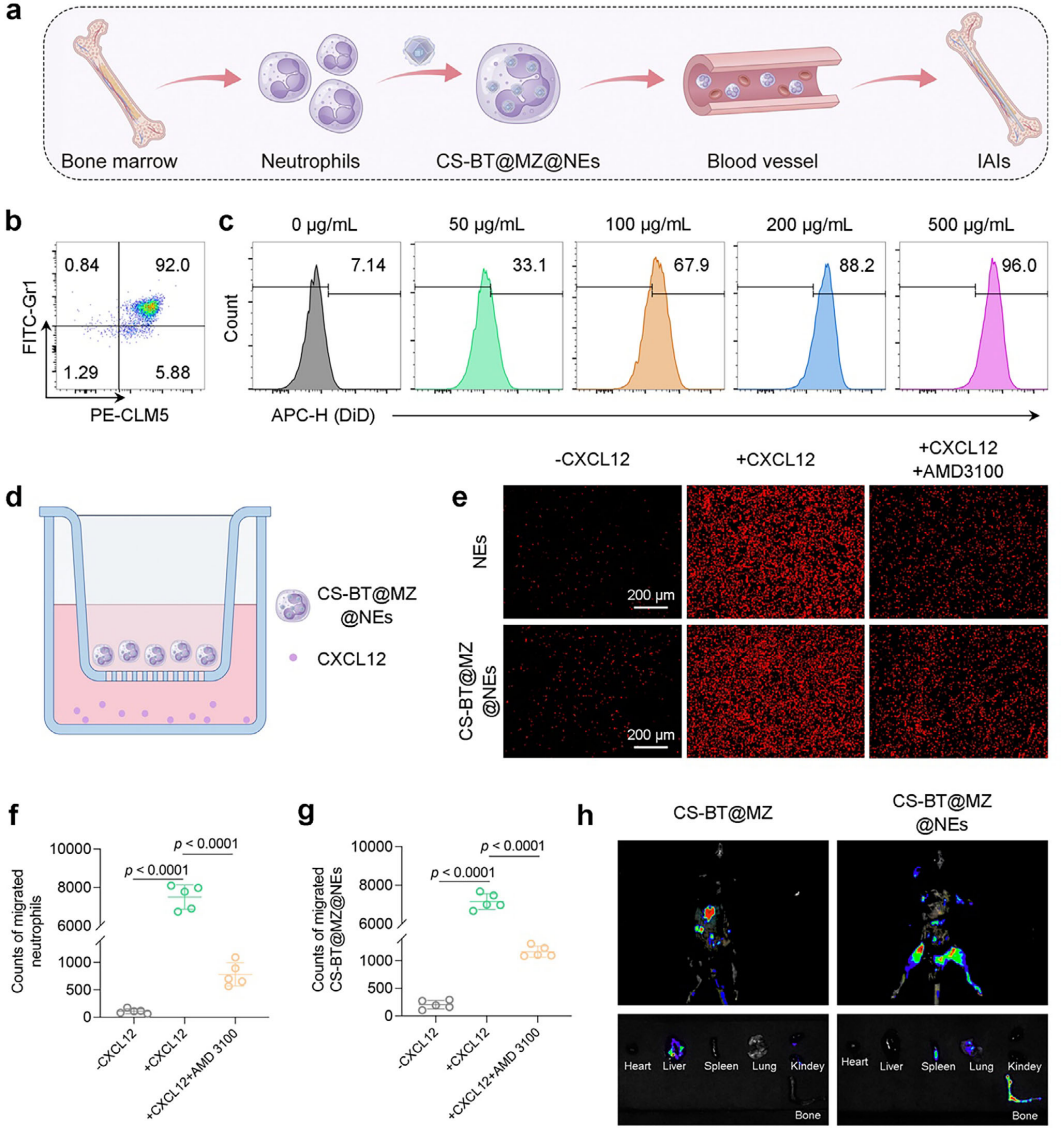
Bone marrow targeting capability of CS‐BT@MZ@NEs. (a) Schematic illustrating neutrophil‐mediated nanoparticle uptake and homing to bone marrow. (b) Flow cytometric analysis of neutrophil purity using FITC anti‐mouse Ly‐6G/Ly‐6C (Gr1) and PE anti‐mouse MAIR‐IV (CLM‐5) antibodies. (c) Representative flow cytometry plots of DiD^+^ neutrophils under different conditions. (d) Schematic of the Transwell migration assay with CXCL12 in the lower chamber. (e) Fluorescence images of neutrophils or CS‐BT@MZ@NEs (DiD‐labeled) migrating to the lower chamber with or without CXCL12 and AMD3100. (f, g) Quantification of migratory neutrophils and CS‐BT@MZ@NEs using ImageJ. (h) Representative fluorescence images of trunks and major organs at 12 h after intravenous injection of DiR‐CS‐BT@MZ or DiR‐CS‐BT@MZ@NEs. Data are presented as mean ± SD (n = 5 per group), with “n” denoting biologically independent experiments.

Based on this, the present study proposes utilizing neutrophils cultured in vitro for 6 h as a drug delivery tool. By co‐incubating NPs with these high CXCR4‐expressing neutrophils, the aim is to achieve effective nanoparticle loading and leverage the natural migration properties of neutrophils to precisely deliver drugs to the deep bone marrow, thereby overcoming the limitations of traditional drug delivery strategies and providing a novel approach for the treatment of IAIs (Figure 5a). Neutrophils were isolated from bone marrow using density gradient centrifugation and identified via flow cytometry, achieving a purity of approximately 91% (Figure 5b). Subsequently, DiD‐labeled nNPs were co‐cultured with neutrophils for 1 h to assess the degree of NP internalization. The results indicated that the concentration of NPs significantly influenced the uptake efficiency by neutrophils. As the concentration of NPs increased, the fluorescence signal detected in neutrophils progressively intensified. Specifically, the proportion of DiD^+^ neutrophils (DiD‐NPs@NEs) rose from 33.1% at an NP concentration of 50 µg/mL to 96% at 500 µg/mL (Figure 5c). This finding suggests that NP concentration directly affects their internalization efficiency by neutrophils, providing critical insights for optimizing nanoparticle delivery systems.

To evaluate the chemotactic response of neutrophils to CXCL12, a Transwell assay was employed (Figure 5d). Neutrophils loaded with nanoparticles (CS‐BT@MZ@NEs), isolated from bone marrow and cultured for 6 h, were seeded in the upper chamber of the Transwell, while CXCL12 was added to the lower chamber. The experimental results (Figure 5e–g) demonstrated that CS‐BT@MZ@NEs exhibited significant chemotaxis toward CXCL12, efficiently traversing the 3 µm pore membrane and migrating to the lower chamber. After 0.5 h, the number of neutrophils in the lower chamber increased significantly in the presence of CXCL12 compared to the control group without CXCL12, where almost no cell migration was observed. Furthermore, the addition of the CXCR4 receptor antagonist AMD3100 markedly reduced the chemotactic ability of neutrophils toward CXCL12, further confirming the pivotal role of the CXCR4‐CXCL12 axis in the chemotactic process of neutrophils. These findings indicate that CS‐BT@MZ@NEs possess a high sensitivity and chemotactic capability toward CXCL12, providing theoretical support and experimental foundation for their efficient bone marrow delivery in vivo applications. To preliminarily evaluate the in vivo distribution pattern of CS‐BT@MZ@NEs, DiR‐labeled nanoparticles and NEs‐loaded nanoparticles were administered via tail vein injection, and fluorescence imaging was performed at 12 h post‐injection using an in vivo imaging system. The results indicated that the fluorescence signals of DiR‐NPs were predominantly localized in the liver, suggesting a propensity for capture by the reticuloendothelial system (RES). In contrast, the signals of DiR‐CS‐BT@MZ@NEs were significantly reduced in the liver but markedly increased in the spleen, lungs, and bone marrow (Figure 5h).

These findings indicate that neutrophil incorporation alters the in vivo distribution pattern of the nanoparticles at 12 h post‐injection, with reduced hepatic accumulation and enhanced signal in the spleen, lungs, and bone marrow. This result supports the bone marrow‐targeting tendency of CS‐BT@MZ@NEs and provides preliminary in vivo evidence for its delivery advantage in the IAI setting. At the current stage, this delivery strategy is intended to demonstrate the feasibility of neutrophil‐mediated transport in deep infection targeting, whereas its clinical translation will require further optimization of in vivo loading efficiency and safety.

### 2.6 CS‐BT@MZ@NEs‐Induced IAIs Regression and Immune Responses

**FIGURE 6:**
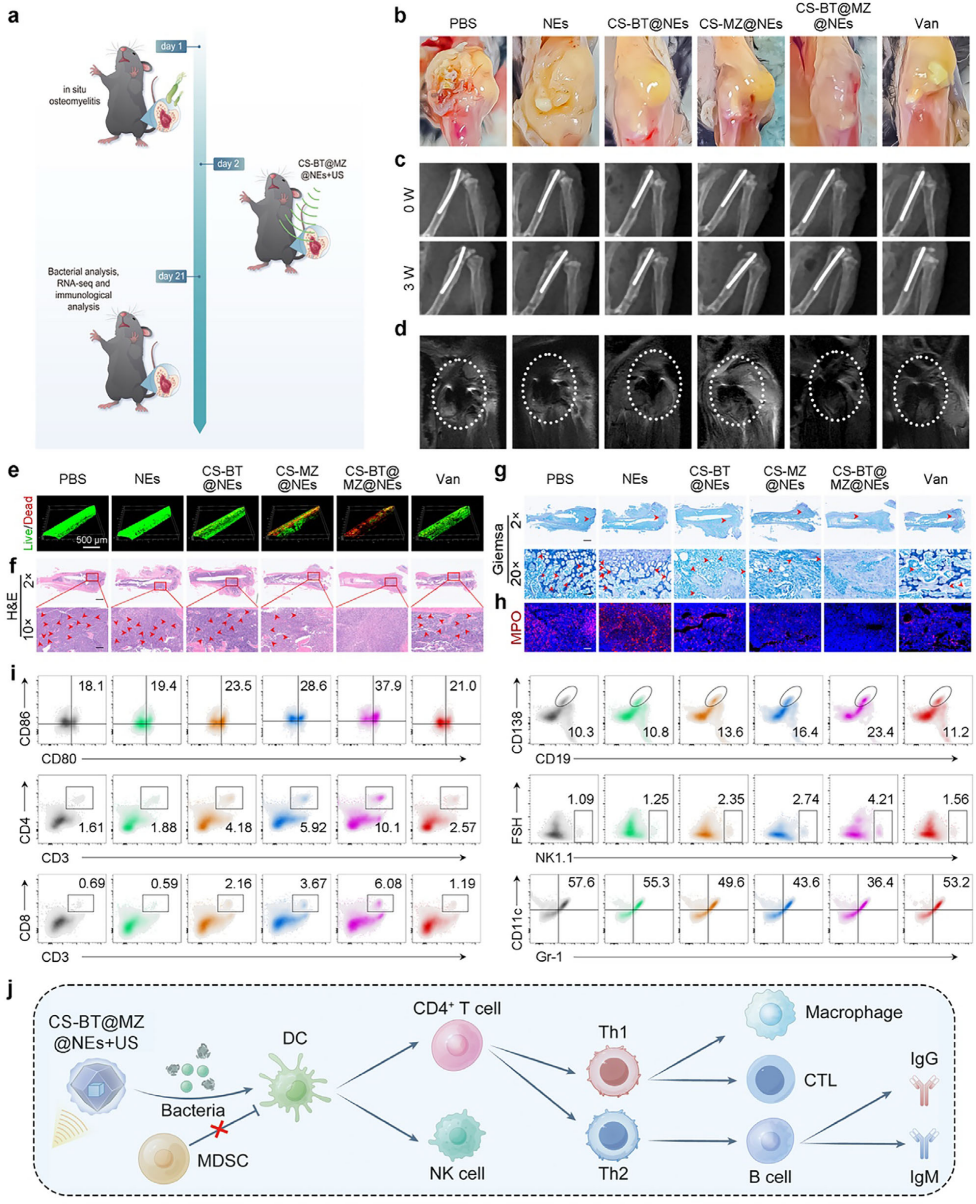
CS‐BT@MZ@NEs “in situ” inhibits bacterial growth and recruits and activates abundant immune cells in IAIs. (a) Schematic illustration of the IAIs model establishment. (b) Digital photos of the infected bone 21 days after the primary surgery. (c) X‐ray imaging of the infected bone. (d) MRI imaging of the infected bone. The white circle indicates the infected bone. (e) Representative images of biofilm CLSM. (f‐h) H&E staining, Gimesa staining, and MPO immunofluorescence images of infected bone. Scale bars: (f) 500 µm (top) and 50 µm (bottom); (g) 500 µm (top) and 50 µm (bottom); (h) 200 µm. (i) Representative flow cytometry plots of mature DCs, CD4^+^ T cells, CD8^+^ T cells, plasmablasts, NK cells, and MDSCs cells in the bone marrow on day 21 after treatments. (j) Schematic representation of the process of CS‐BT@MZ@NEs promoting immunity in vivo. Data are presented as mean ± SD (n = 5 per group), with “n” denoting biologically independent experiments.

The in vitro studies have demonstrated that CS‐BT@MZ@NEs can activate the STING pathway and enhance antigen presentation mediated by DCs. Based on these findings, we hypothesize that CS‐BT@MZ@NEs may induce localized inflammation in immunosuppressive IAIs and bolster the host's immune defense mechanisms. To test this hypothesis, we evaluated knee abscess size, microbial changes, and radiological features in an IAIs model (Figure 6a). The experimental results indicated that mice in the control (PBS) group exhibited severe knee swelling and suppuration, whereas the CS‐BT@MZ@NEs group showed a significant reduction in knee abscess size with no apparent signs of infection (Figure 6b). X‐ray analysis further revealed that chronic biofilm infection led to significant periosteal reactions and osteolysis, while the CS‐BT@MZ@NEs treatment experienced minimal bone destruction, preserving bone architecture (Figure 6c). Micro‐CT analysis demonstrated that the CS‐BT@MZ@NEs group significantly reduced peri‐implant osteolysis, maintaining higher bone mineral density (BMD) and bone volume fraction (BV/TV) compared to other groups (Figure S8a,b). Additionally, MRI indicated a marked decrease in T2 signal in the CS‐BT@MZ@NEs group, reflecting a substantial reduction in infection severity (Figure 6d). To further validate the antibacterial efficacy, CLSM was performed on harvested implants. The results showed a significant reduction in bacterial colonies in the CS‐BT@MZ@NEs treatment group (Figure 6e). Histological analysis further demonstrated a marked decrease in neutrophils and bacterial infiltration in this treatment group (Figure 6f–h).

To further elucidate the intrinsic antibacterial mechanism of CS‐BT@MZ@NEs, we conducted a comprehensive analysis of the immune cell composition in bone marrow, focusing on the phenotype of infection‐associated macrophages (IAMs) and the dynamic changes in immune cells. M1 phenotype IAMs are crucial effector cells for bacterial clearance, primarily through phagocytosis and antigen‐dependent mechanisms. The results demonstrated that treatment with CS‐BT@MZ@NEs significantly polarized IAMs in mouse bone marrow toward the M1 phenotype. Immunofluorescence staining further confirmed this phenomenon, showing a marked increase in the expression of iNOS (a marker of the M1 phenotype) and a notable decrease in CD206 (a marker of the M2 phenotype) (Figure S9). Further analysis of the bone marrow immune cell composition revealed that CS‐BT@MZ@NEs treatment significantly enhanced the infiltration of key drivers of antibacterial immunity (Figure 6i; Figure S10). Notably, this treatment significantly increased the number of M1 macrophages (CD86^+^CD206^−^) (Figure S11). Additionally, the proportion of mature DCs (CD80^+^CD86^+^) sharply increased in the CS‐BT@MZ@NEs group. Previous studies have indicated that an increased proportion of mature DCs can substantially enhance pathogen‐directed adaptive immune responses [45, 46, 47]. Similarly, we hypothesize that as a bacterial therapeutic agent, the increase in DCs may significantly improve antigen presentation efficiency, thereby enhancing bacteria‐specific immune responses. Further flow cytometry analysis showed a significant increase in T cell infiltration in the CS‐BT@MZ@NEs group, including CD4^+^ helper T cells (CD45^+^CD3^+^CD4^+^) and CD8^+^ cytotoxic T cells (CD45^+^CD3^+^CD8^+^). The increase in these T cells is crucial for the adaptive immune response of the infected host. CD4^+^ helper T cells amplify the antibacterial effect by regulating the function of other immune cells, while CD8^+^ T cells directly mediate the clearance of bacterial infection sites.

Protective humoral immunity depends on the differentiation of B cells into antibody‐producing plasma cells and memory B cells that respond rapidly upon pathogen re‐exposure [48, 49]. CS‐BT@MZ@NEs significantly increased the proportion of plasma cells (CD45^+^CD19^+^CD138^+^) in infected bone tissue, indicating enhanced humoral immune activation. In addition, because invading pathogens may evade host defense by residing within host cells, the increased recruitment of NK cells (CD45^+^NK1.1^+^) and CD8^+^ T cells after CS‐BT@MZ@NEs treatment suggests strengthened cellular immune surveillance and clearance of persistent infection. Together, these results highlight the ability of CS‐BT@MZ@NEs to reinforce both humoral and cellular antibacterial immunity.

A hallmark of the IME is the significant infiltration of MDSCs in the bone marrow [12, 50]. To evaluate the regulatory effect of CS‐BT@MZ@NEs on the immunosuppressive IME, we analyzed the infiltration of MDSCs. The results indicated a significant reduction in the proportion of CD45^+^Gr1^+^ MDSCs in the CS‐BT@MZ@NEs‐treated group. Given that extensive infiltration of MDSCs leads to T cell dysfunction and impedes the effector function of immune cells, the depletion of MDSCs induced by CS‐BT@MZ@NEs is considered a crucial mechanism for enhancing the efficacy of bacterial immunotherapy. Inspired by these findings, we further examined the levels of cytokines and antibodies in the infected bone IME to explore their roles in driving antibacterial immunity. The results showed that mice treated with CS‐BT@MZ@NEs exhibited significantly elevated levels of pro‐inflammatory cytokines, such as IFN‐β, CXCL10, and IL‐12, while anti‐inflammatory cytokines, like IL‐10, were reduced (Figure S12a). These results further confirm that CS‐BT@MZ@NEs can elicit a robust antibacterial immune response in the low‐immunogenicity IAIs. Inducing humoral responses against bacterial antigens exposed during infection control is an important aspect of antibacterial host defense. In the present system, CS‐BT@MZ@NEs promoted biofilm disruption and bacterial damage, which may facilitate the exposure and presentation of bacterial antigens. Consistently, elevated serum IgM and IgG levels were observed after treatment (Figure S12b), supporting enhanced humoral immune activation in IAIs.

In summary, CS‐BT@MZ@NEs can trigger potent innate and adaptive antibacterial immune responses in IAIs. The innate immune response is achieved through the STING pathway activated by CS‐BT@MZ@NEs, including macrophage M1 polarization and DCs maturation; the adaptive immune response is co‐mediated by CS‐BT@MZ@NEs assisted SDT, CS‐BT@MZ@NEs‐induced protective antibody‐driven humoral immunity, and T cell immunity activated by APCs. These immune responses demonstrate that CS‐BT@MZ@NEs can effectively combat bacterial infections. Moreover, pathological analysis of major organs revealed that CS‐BT@MZ@NEs treatment did not induce significant systemic side effects (Figure S13), further supporting its potential as a safe and effective therapeutic strategy.

**FIGURE 7:**
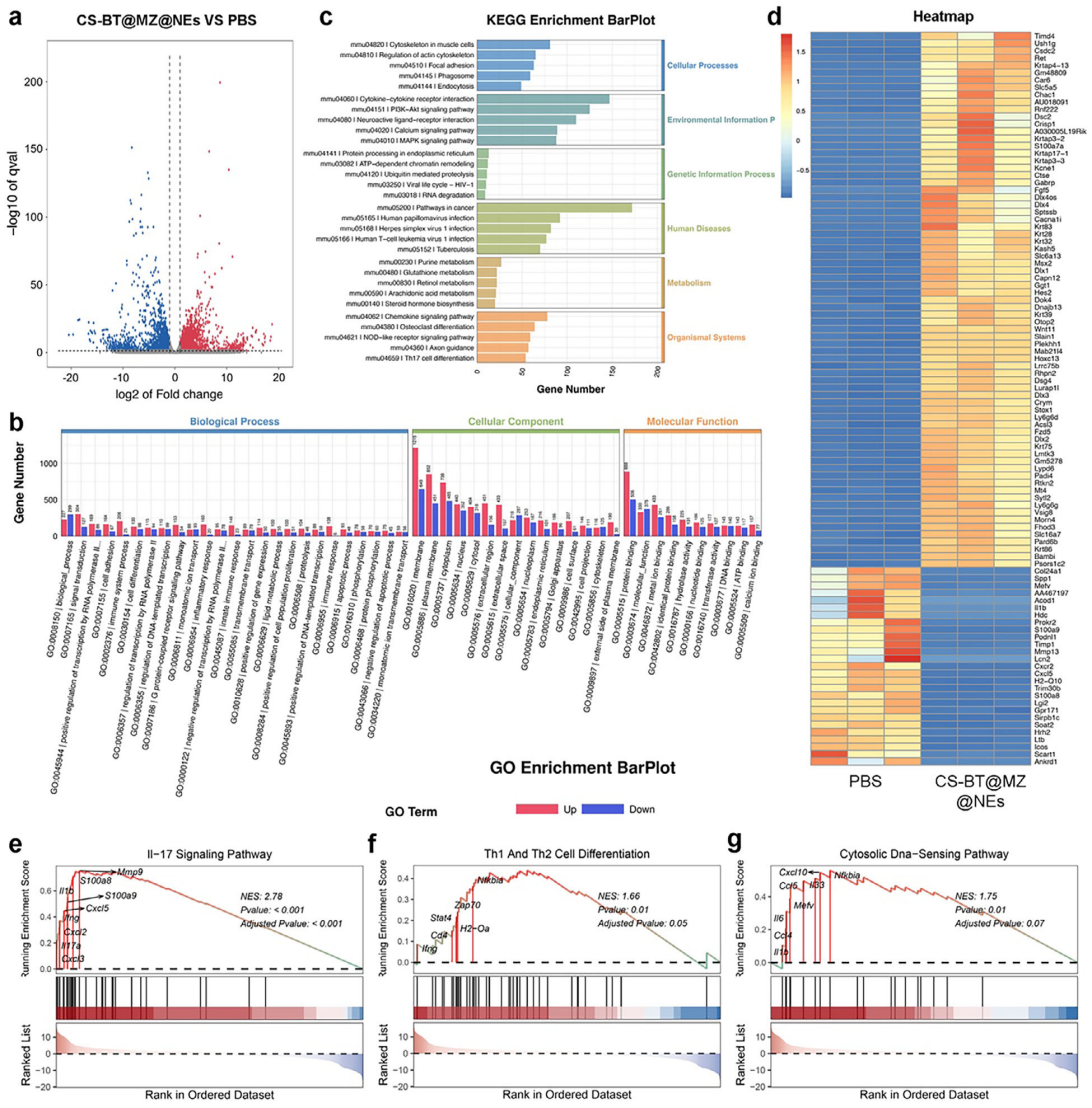
Transcriptomic analysis of CS‐BT@MZ@NEs‐mediated anti‐biofilm immunotherapy. (a) Volcano plot of differentially expressed genes. (b) GO enrichment. (c) KEGG pathway enrichment. (d) Heatmap of regulated genes. (e–g) GSEA of representative immune pathways after CS‐BT@MZ@NEs treatment. Ticks indicate gene rank; NES, normalized enrichment score.

To comprehensively elucidate the anti‐biofilm mechanism of CS‐BT@MZ@NEs, we conducted a transcriptomic analysis on infected tissues from mice treated with either PBS or CS‐BT@MZ@NEs. The results revealed that compared to the biofilm group, treatment with CS‐BT@MZ@NEs led to the upregulation of 1056 genes and downregulation of 1138 genes (Figure 7a). GO analysis indicated a significant enrichment of pathways related to the immune system, particularly those involved in immune system processes and innate immune responses (Figure 7b), suggesting a strong association between CS‐BT@MZ@NEs and immune‐related pathways, thereby triggering a potent anti‐biofilm immune response. Furthermore, KEGG pathway analysis corroborated these findings (Figure 7c). CS‐BT@MZ@NEs activated the STING pathway, initiating a cascade of immune responses, including the upregulation of interferon‐stimulated genes (e.g., Ifng), pro‐inflammatory cytokines (e.g., Il1b and Il17a), leukocyte recruitment chemokines (e.g., Cxcr2, Cxcr5, Cxcl3), and T cell and DCs activation markers (e.g., CD4, H2‐Q10) (Figure 7d). The upregulation of these immune effector molecules indicates that CS‐BT@MZ@NEs effectively promotes the recruitment and activation of various immune cells. Additionally, GSEA analysis demonstrated that the activity of the IL‐17 signaling pathway, Th1 and Th2 cell differentiation pathways, and cytosolic DNA sensing pathways were significantly enhanced in the CS‐BT@MZ@NEs treatment group (Figure 7e–g). These changes suggest that key co‐stimulatory signaling pathways were effectively stimulated, further amplifying the anti‐biofilm immune response.

Thus, the CS‐BT@MZ@NEs nanoplatform efficiently activate the STING pathway in vivo, inducing the expression of interferon‐stimulated genes, amplifying pro‐inflammatory signaling, and promoting the recruitment and activation of various immune cells, ultimately achieving a robust anti‐biofilm effect. This discovery provides critical theoretical and practical guidance for the immunotherapy of IAIs.

### 2.7 CS‐BT@MZ@NEs‐Inflamed Transferable Antibacterial Immune Responses

**FIGURE 8:**
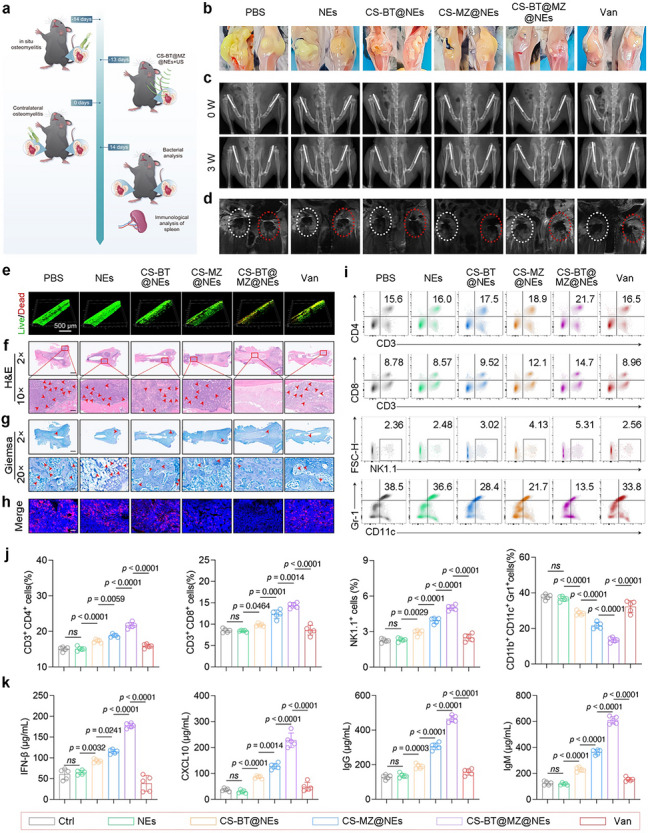
CS‐BT@MZ@NEs inflames systemic antibacterial immune responses against contralateral IAIs. (a) Scheme and timeline of the experimental design to evaluate the systemic immune responses triggered by CS‐BT@MZ@NEs. (b) Digital photos of the infected bone in 14 days after the construction of the contralateral osteomyelitis model by secondary surgery. (c) X‐ray imaging of the infected bone. (d) MRI imaging of both sides of the infected bone. The white and red circle indicate the first and secondary IAIs, respectively. (e) Representative images of biofilm CLSM. (f‐h) H&E staining, Gimesa staining, and MPO immunofluorescence images of infected bone. Scale bars: (f) 500 µm (top) and 50 µm (bottom); (g) 500 µm (top) and 50 µm (bottom); (h) 200 µm. (i,j) Representative flow cytometry plots and quantification of CD4^+^ T cells, CD8^+^ T cells, NK1.1 cells, and MDSCs cells in the spleens 14 days after the secondary IAIs. (k) IFN‐β, CXCL10, IgG, and IgM concentrations in the serum. Data are presented as mean ± SD (n = 5 per group), with “n” denoting biologically independent experiments.

Uncontrolled localized bacterial infections can progressively lead to fatal bacteremia and damage to distant organs [51, 52]. Additionally, infection‐associated immunosuppression increases the host's susceptibility to secondary infections [53]. To evaluate whether CS‐BT@MZ@NEs nanoplatform can prevent the formation of distant infection foci by inducing an effective systemic antibacterial immune response, we established a contralateral IAIs model in mice (Figure 8a). The experimental procedure involved initially establishing an in situ IAIs model and immunizing the mice with CS‐BT@MZ@NEs, followed by creating another IAIs model in the contralateral knee joint. Fourteen days post‐model establishment, a multidimensional analysis of the contralateral infected knee joint was conducted, including knee abscess size, microbial changes, radiological features, and Giemsa staining. The results demonstrated a significant reduction in infection severity in mice treated with CS‐BT@MZ@NEs, evidenced by markedly reduced knee joint swelling (Figure 8b). X‐ray and MRI results showed diffuse edema and liquefactive necrosis in the joint cavity and surrounding muscles of control mice, whereas the CS‐BT@MZ@NEs treatment group exhibited significantly alleviated infectious lesions (Figure 8c,d). CLSM images indicated a substantial decrease in bacterial burden in the immunized group of mice (Figure 8e). Pathological sections further supported these findings: control mice displayed extensive neutrophilic and bacterial infiltration, while the tissue morphology in the CS‐BT@MZ@NEs treatment group was nearly normal (Figure 8f–h). These results suggest that in situ inoculation with CS‐BT@MZ@NEs can significantly mitigate infection severity in the contralateral IAIs model.

Splenic immune responses were next examined to investigate the mechanism underlying this phenomenon. As a central lymphoid organ in systemic host defense, the spleen coordinates antigen‐driven immune activation. The results showed that CS‐BT@MZ@NEs induced marked splenic immune activation in IAIs mice, involving both innate and adaptive immunity (Figure 8i,j). This was accompanied by increased pro‐inflammatory cytokine production and activation of adaptive immune cells, supporting systemic antibacterial immunity. Further analysis revealed that mice immunized with CS‐BT@MZ@NEs exhibited peak infiltration of CD4^+^ and CD8^+^ T cells, as well as NK cells, within splenic tissues. The expansion of CD8^+^ T cells in lymphoid organs is a hallmark of enhanced systemic immunity, representing a significant boost in cellular immunity. Concurrently, CS‐BT@MZ@NEs effectively inhibited the influx of regulatory immune cells, such as MDSCs, thereby mitigating the immunosuppressive effects induced by bacterial infection. This immune cell profiling collectively validates that CS‐BT@MZ@NEs can induce potent systemic humoral and cellular immunity, preventing bacterial dissemination. To comprehensively characterize the host immune response, we conducted ELISA analyses of cytokine and antibody levels in serum (Figure 8k). The results indicated a marked increase in pro‐inflammatory cytokine levels and a significant decrease in anti‐inflammatory cytokine levels in the CS‐BT@MZ@NEs treatment group, suggesting that CS‐BT@MZ@NEs can trigger a robust pro‐inflammatory response, thereby amplify the antimicrobial immune response and achieving therapeutic effects. Additionally, the serum IgM and IgG responses in CS‐BT@MZ@NEs immunized mice were significantly enhanced, surpassing those of other experimental groups, further demonstrating a potent humoral immune response.

In short, CS‐BT@MZ@NEs nanoplatform, through in situ immunization, not only effectively control localized infections but also significantly reduce the severity of distant infection foci by enhancing systemic immune responses. This discovery provides crucial support for the application of CS‐BT@MZ@NEs in immunotherapy and demonstrates their immense potential in IAIs treatment.

### 2.8 CS‐BT@MZ@NEs‐Conferred Long‐Lasting Protective Immunity against IAIs

**FIGURE 9:**
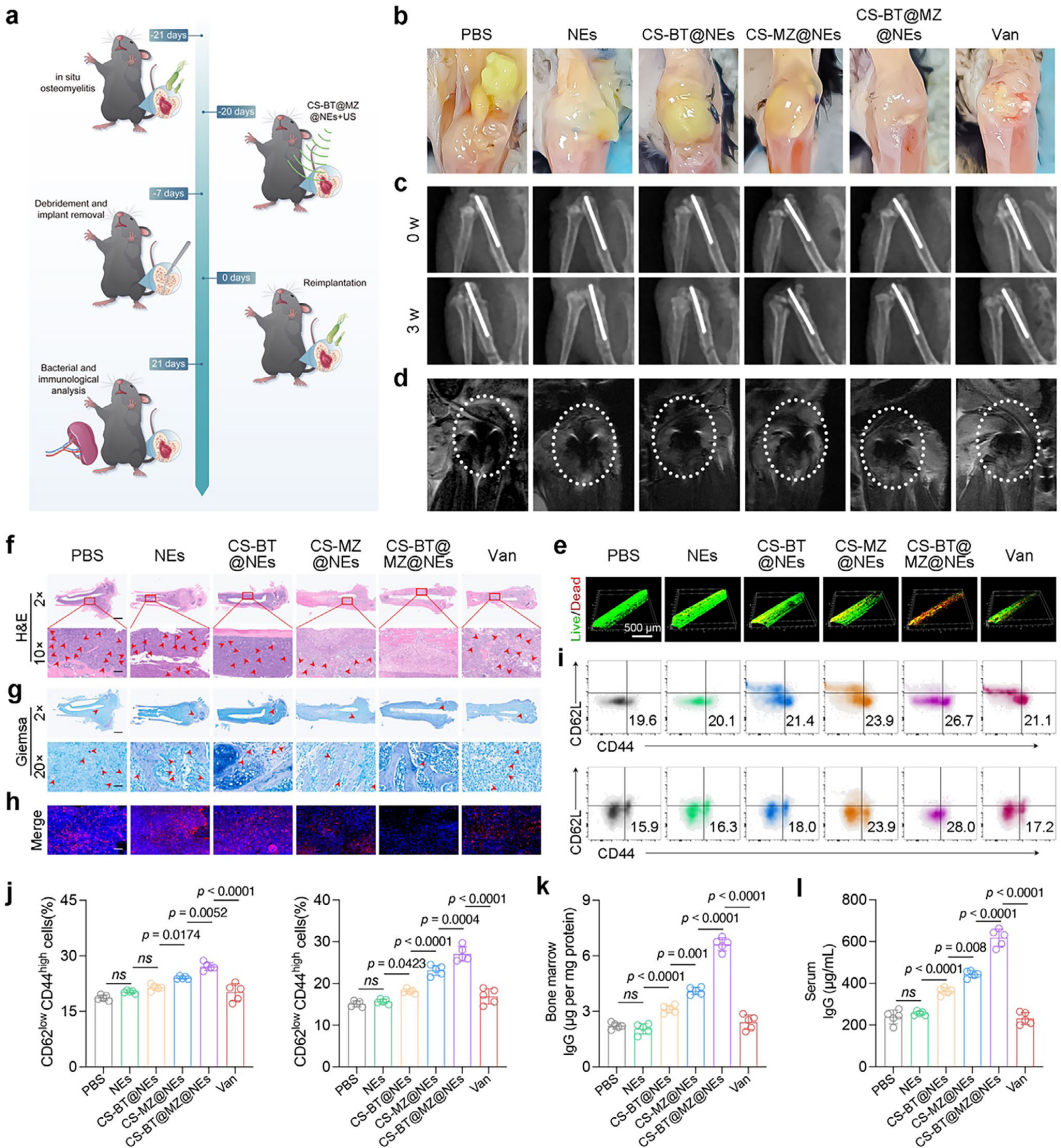
CS‐BT@MZ@NEs elicits the long‐term memory immune response to prevent IAIs recurrence. (a) Schematic illustration of the post‐surgery CS‐BT@MZ@NEs in the recurrent IAIs model. (b) Digital photos of the rechallenged infected bone 21 days after the reimplantation and *MRSA* injection. (c) X‐ray imaging of the reinfected bone. (d) MRI imaging of the reinfected knee. The white circle indicates the reinfected knee. (e) Representative images of biofilm CLSM. (f‐h) H&E staining, Gimesa staining, and MPO immunofluorescence images of infected bone. Scale bars: (f) 500 µm (top) and 50 µm (bottom); (g) 500 µm (top) and 50 µm (bottom); (h) 200 µm. (i,j) Representative flow cytometry plots and quantification of CD4^+^ memory T cells and CD8^+^ memory T cells in the spleens 21 days after the reimplantation and MRSA injection. (k,l) IgG concentrations in the bone marrow and serum of rechallenged mice. Data are presented as mean ± SD (n = 5 per group), with “n” denoting biologically independent experiments.

To investigate the potential of CS‐BT@MZ@NEs nanoplatform in eliciting an antimicrobial immune recall response in a prophylactic setting, we further examined their ability to prevent infection recurrence following primary immune clearance. To simulate clinically relevant scenarios, we initially eradicated established IAIs in mice using CS‐BT@MZ@NEs immunotherapy, followed by re‐challenging these mice with *MRSA* (Figure 9a). The results demonstrated that mice pre‐immunized with CS‐BT@MZ@NEs exhibited significant anti‐infective capabilities upon bacterial re‐challenge, with mild infection symptoms and a marked reduction in bacterial load (Figure 9b–h). Notably, the high antigenic variability and microbiome heterogeneity in infections often limit the immune memory response of current antimicrobial therapies to specific pathogens [54, 55]. However, flow cytometry analysis revealed that CS‐BT@MZ@NEs treatment significantly expanded the populations of CD4^+^ memory T cells (CD4^+^CD44^high^CD62L^low^) and CD8^+^ memory T cells (CD8^+^CD44^high^CD62L^low^) in the spleen compared to controls (Figure 9i,j). Furthermore, to assess humoral memory‐associated antibody responses after bacterial rechallenge, we analyzed IgG levels in the serum and bone marrow of pre‐treated mice. The results showed increased IgG levels in the CS‐BT@MZ@NEs group (Figure 9k,l), supporting enhanced humoral immune protection in the recurrent MRSA infection setting [56, 57].

In summary, CS‐BT@MZ@NEs not only demonstrated significant therapeutic effects in primary infections but also enhanced host resistance to infection recurrence by inducing durable memory T cell responses and antibody production. These findings underscore the potential application of CS‐BT@MZ@NEs in combating infection recurrence and sustaining immune defense.

## 3 Conclusions

This study elucidates the antibacterial mechanism, immunomodulatory functions, and Golgi pH‐regulating role of the CS‐BT@MZ@NEs nanoplatform for the treatment of IAIs. By leveraging neutrophil hitchhiking, the nanoplatform achieves efficient bone marrow‐targeted delivery to deep infection sites. At the lesion site, CS‐BT@MZ@NEs disrupts bacterial biofilms under US stimulation, promotes antigen exposure, and modulates Golgi pH through its Golgi‐targeting capability, thereby enhancing STING signaling. Golgi acidification facilitates STING oligomerization and TBK1 activation, leading to interferon‐ and pro‐inflammatory cytokine‐associated immune activation. This process promotes macrophage M1 polarization and DC maturation, thereby amplifying innate immunity. Meanwhile, CS‐BT@MZ@NEs also enhances adaptive immune responses by reducing MDSC infiltration and increasing CD4^+^ and CD8^+^ T‐cell infiltration. In addition, treated mice exhibit increased memory T‐cell populations and elevated IgG levels, supporting improved immune protection against bacterial reinfection. Overall, CS‐BT@MZ@NEs provides an integrated strategy for IAI treatment by combining antibacterial activity, Golgi pH modulation, and immune memory induction. From a translational perspective, the neutrophil‐hitchhiking strategy described here should be regarded primarily as a proof‐of‐concept approach for exploiting the intrinsic migratory behavior of neutrophils to enhance delivery across the bone marrow–blood barrier. Rather than relying on extensive ex vivo manipulation, future optimization may focus on improving in situ interactions between nanoparticles and endogenous circulating neutrophils under inflammatory conditions. Further studies will be required to evaluate biosafety, delivery efficiency, and clinical applicability in more realistic translational settings.

## Conflicts of Interest

The authors declare no conflicts of interest.

## Supporting information




**Supporting File**: advs75623‐sup‐0001‐SuppMat.docx.

## Data Availability

The data that support the findings of this study are available from the corresponding author upon reasonable request.
