# Supplementary material for: Targeting Golgi–STING Signaling to Reprogram Innate and Adaptive Immunity for the Treatment of Implant‐Associated Infections
Source: Adv Sci (Weinh). 2026 May 10;13(43):e75623. doi: 10.1002/advs.75623 (PMC13335843; doi:10.1002/advs.75623)
Supplement: Supplementary file 1 — Supporting File: advs75623‐sup‐0001‐SuppMat.docx. [file ADVS-13-e75623-s001.docx]

**Targeting Golgi–STING Signaling to Reprogram Innate and Adaptive Immunity for the Treatment of Implant-Associated Infections**

*Shicheng Huo^1, #^, Naifeng Zhu^2, #^, Zhuocheng Lyu^2, #^, Yifei Liu^1, #^, Zhenjiang Xu^1^, Chun Zhou^2,^ *, Yaochao Zhao^2,^ *, Changgui Shi^1,^ **

^1^ Department of Orthopedic Surgery, The Spine Surgical Center, Second Affiliated Hospital of Naval Medical University, Shanghai, 200003, China

^2^ Department of Joint Replacement, Sports Medicine, and Trauma,Department of Orthopedics，Renji Hospital, Shanghai Jiaotong University School of Medicine, Shanghai, 200001, China

*Corresponding authors. E-mail: Changgui Shi (charlieshi@smmu.edu.cn), Yaochao Zhao (zyc308@126.com), Chun Zhou (zoucnu@163.com)

Shicheng Huo^#^, Naifeng Zhu^#^, Zhuocheng Lyu^#^, and Yifei Liu^#^ contributed equally to this work.

**Experimental Section**

***Mice:*** All animal experiments were approved by the Animal Ethics Committee of the Second Hospital of Naval Medical University (2023-564). All manipulations and surgical procedures followed approved guidelines.

***Bacterial strains and cell lines:*** The bacteria used in this study were *MRSA* strains (ATCC 43300), obtained from the American Type Culture Collection and propagated in TSB medium (Hopebio). Raw 264.7 cells were provided by Shanghai Institute of Cell Biology and maintained in DMEM medium supplemented with 10% FBS and 1% penicillin-streptomycin. The isolation and culture of mouse bone marrow-derived macrophages (BMDMs) and bone marrow-derived dendritic cells (BMDCs) were performed according to previously established protocols. ^[1]^

***Preparation of nanoparticles***: *Synthesis of BaTiO3 Nanoparticles*: BaTiO_3_ nanoparticles were synthesized using a solvothermal method with Ba(OH)_2_·H_2_O (98%, Sigma-Aldrich) and Ti[O(CH_2_)_3_CH_3_]_4_ (Tibutoxide, 97%, Sigma-Aldrich) as starting materials. In a typical preparation, 17.018 g (50 mmoL) of Tibutoxide was mixed with 20 mL of ethanol (analytical grade), followed by the addition of 7 mL of ammonium hydroxide solution (25%, Sigma-Aldrich) to the mixture. Concurrently, 14.204 g (75 mmoL) of Ba(OH)_2_·H_2_O was dissolved in 25 mL of deionized water to prepare a clear barium hydroxide solution. This solution was then added to the mixed solution. The resulting mixture was transferred to a 100 mL Teflon-lined stainless-steel autoclave and subjected to thermal treatment at 200 °C for 48 h. Upon completion of the reaction, the precipitate was centrifuged and washed 2-3 times with acetic acid and anhydrous ethanol. The precipitate was then dried in an oven at 80 °C for 24 h. Finally, the dried precipitate was placed in an alumina crucible and heated in a tube furnace in air at 800 °C for 10 h, followed by natural cooling to room temperature. Post-annealing, the agglomerated powder was ground in a mortar to produce fine BaTiO_3_ (BT) powder.

*Preparation of BaTiO3@CS Nanoparticles*: Dissolve 0.1 g of chondroitin sulfate (CS) in 20 mL of deionized water. Subsequently, introduce 100 mg of BaTiO_3_ nanoparticles into the CS aqueous solution and stir at room temperature for 24 h. Finally, collect the nanoparticles by centrifugation at 13,000 rpm, and wash them 2-3 times with deionized water to obtain BaTiO_3_@CS (CS-BT) nanoparticles.

*Preparation of Mn-ZIF-8@CS Nanoparticles*: Initially, 0.149g of Zn(NO_3_)_2_·6H_2_O and 0.125g of Mn(NO_3_)_2_ were dissolved in 11.3 mL of methanol and stirred at room temperature for 20 min. Subsequently, 0.656g of 2-methylimidazole was dissolved in 11.3 mL of methanol and gradually added dropwise to the nanoparticle mixture solution, with stirring continued for an additional 2 h. The nanoparticles were then collected by centrifugation at 13,000 rpm and washed 2-3 times with deionized methanol to obtain Mn-ZIF-8 nanoparticles. Finally, Mn-ZIF-8@CS nanoparticles were synthesized using the method employed for BaTiO_3_@CS nanoparticles.

*Synthesis of BaTiO_3_@Mn-ZIF-8@CS Nanoparticles*: Initially, 0.149g of Zn(NO_3_)_2_·6H_2_O and 0.125g of Mn(NO_3_)_2_ were dissolved in 11.3 mL of methanol, followed by the addition of 0.1 g of BaTiO_3_ nanoparticles, with stirring at room temperature for 20 min. Subsequently, 0.656 g of dimethylimidazole was dissolved in 11.3 mL of methanol and gradually added dropwise to the nanoparticle mixture, with continuous stirring for 2 h. The nanoparticles were then collected by centrifugation at 13000 rpm and washed 2-3 times with deionized water to obtain BaTiO_3_@Mn-ZIF-8 (BT@MZ) nanoparticles. Finally, BaTiO_3_@Mn-ZIF-8@CS (CS-BT@MZ) nanoparticles were synthesized using the method for preparing BaTiO_3_@CS nanoparticles.

*Preparation of BaTiO_3_@Mn-ZIF-8@CS-FITC (DiD) Nanoparticles*: Initially, 0.1 g of BaTiO_3_@Mn-ZIF-8 was ultrasonically dispersed in 10 mL of deionized water and stirred at room temperature for 5 min. Subsequently, 0.01 g of FITC (DiD) fluorescent dye was added to the solution, and the mixture was stirred for an additional 8 h. The nanoparticles were then collected by centrifugation at a speed of 13,000 rpm and washed 2-3 times with deionized water to obtain BaTiO_3_@Mn-ZIF-8@CS-FITC (DiD) nanoparticles. The preparation methods for BaTiO_3_@CS-FITC (DiD) and Mn-ZIF-8@CS-FITC (DiD) nanoparticles were identical.

***Characterization***: The synthesized various nanoparticles are characterized using Transmission Electron Microscopy (TEM), X-ray diffraction (XRD), XPS (ESCALAB 250Xi, Thermo Fisher, USA) and FTIR (Magna-IR 750, Thermo Fisher, USA) analysis to confirm their structural and morphological properties. To investigate the radical mechanism within the ultrasonic Fenton piezoelectric system, we conducted EPR radical trapping experiments to ascertain the contribution of active species. We employed TBA, Na_2_C_2_O_4_, BQ, and TEMP as quenchers for •OH, h+, •O_2_^-^, and ^1^O_2_, respectively, with DMPO (10 mM) serving as the radical trapping agent, all at a concentration of 10 mM. The output voltage was measured using a digital oscilloscope (DHO1000, China) under US triggering conditions. The experimental parameters were configured as follows: a center frequency of 1 MHz, a pulse repetition frequency of 1000 Hz, a pressure intensity of 30 mW/cm², and a duty cycle of 50%. The Piezo Force Microscope (PFM) utilizes the inverse piezoelectric effect of piezoelectric materials to detect the piezoelectric response of the materials. Prior to testing, the sample solution is dropped onto a silicon chip, dried, and then characterized. To investigate the radical mechanism of the entire system in US. KPFM (Bruker, Dimension Icon) was used to detect the surface potential distribution. Nano Scope Analysis software (1.40r1, Bruker, USA) was used to analyze and obtain the surface potential data. The surface potential data was then fitted by Gaussian fitting, and the average surface potential was finally calculated according to the weighted average.

***Density Functional Theory (DFT) Calculations:*** To elucidate the interfacial properties of the TiO and Mn, Zn heterojunctions, DFT simulations were conducted using the Vienna Ab initio Simulation Package (VASP). The simulations employed the Generalized Gradient Approximation (GGA) exchange-correlation functional within the Perdew-Burke-Ernzerhof (PBE) framework. The electronic structure characteristics of TiO and Mn, Zn conductors were determined through band structure and density of states (DOS) diagrams derived from first-principles DFT calculations.

***US treatment:*** US irradiation was applied using an Intelect Mobile Ultrasound Device (Chattanooga 2776, DJO Group, USA) at a frequency of 1.0 MHz, an intensity of 1.5 W/cm², and a duty cycle of 50%. Unless otherwise indicated, the same ultrasound parameters were used in all relevant experiments. These parameters were chosen based on previous reports to achieve effective antimicrobial activity while maintaining acceptable biocompatibility.

***In vitro antimicrobial assay:*** Thaw the frozen MRSA cultures and incubate them overnight in tryptic soy broth (TSB) at 37 °C with shaking. Subsequently, dilute the resulting culture 1:10000 and continue incubation until it reaches the logarithmic growth phase. Introduce 500 μL of the bacterial solution in the logarithmic phase (concentration of 1 × 10^7^ CFU/mL) onto sterilized titanium discs placed in a 24-well plate. Incubate at 37 °C for 24 h to facilitate the formation of mature biofilms. Various therapeutic agents, including PBS (control), CS-BT, CS-MZ, CS-BT@MZ, and vancomycin, are then introduced to the mature biofilms and incubated for 2 h. The biofilm formation capacity is assessed as follows: Dispense 360 μL of diluted MRSA solution (10^9^ CFU/mL) onto the titanium discs in the 24-well plate, add 40 μL of the respective treatment system, and incubate the mixture for 2 h. Subsequently, subject these plates to 5 min of ultrasonic irradiation (1.5 W/cm^2^, 40 kH) or leave them without irradiation, followed by a 24-h incubation. Evaluate the antibiofilm efficacy of different treatments using live/dead staining, crystal violet staining, and scanning electron microscopy: Employ the LIVE/DEAD BacLight Bacterial Viability Kit according to the manufacturer's instructions. Additionally, stain the biofilms with 1% crystal violet for 15 min. Rinse the titanium discs twice with deionized water and dissolve them in 96% ethanol. Measure the optical density at a wavelength of 595 nm using a microplate reader to assess the biofilm mass.

Furthermore, the biofilm was rinsed with sterile PBS and resuspended in 1 mL of physiological saline. Congo red was then added to the solution to achieve a final concentration of 40 µg/mL. The membrane was subsequently incubated at 37 °C and agitated at 200 rpm using a thermostatic shaker for 2 h. The absorbance of the supernatant was measured at a wavelength of 490 nm. During detection, 1 mL of physiological saline containing Congo red served as the control. The formula for calculating the Congo red binding rate is as follows: Congo red binding rate (%) = 100 - [(OD of test supernatant × 100) / OD of control group%]. Additionally, dsDNA was extracted from the treated biofilm using a polymer-based circulating free DNA enrichment kit (Analytik Jena AG, Jena, Germany) according to the manufacturer's instructions. The concentration of dsDNA was quantified using a NanoDrop spectrophotometer, with each experimental group undergoing at least five replicates.

Subsequent to this, the biofilms were harvested post-rinsing with sterile PBS, and their bacterial viability was quantified via a CFU assay, while the integrity of their bacterial membranes was assessed through live/dead staining. In brief, the bacterial suspension was subjected to three PBS washes before the addition of a dual dye comprising SYTO9 and PI. After a 15-minute co-incubation in the dark, the samples underwent three further PBS washes and were then analyzed by flow cytometry. The treated bacterial samples were incubated with propidium iodide (PI) for 30 min. The stained solution was also examined using flow cytometry. Subsequently, the fluorescence intensity of these samples was measured using a microplate reader, with excitation and emission wavelengths set at λex = 535 nm and λem = 615 nm, respectively. Bacteria subjected to various treatments were stained with DCFH-DA, followed by analysis using flow cytometry and a microplate reader to assess intracellular ROS levels.

For TEM analysis, bacterial samples were fixed overnight at 4 °C in a 2.5% glutaraldehyde solution and subsequently rinsed three times with PBS. The samples were then dehydrated using a graded ethanol series, infiltrated with embedding medium, and embedded. Ultrathin sections of the embedded samples were prepared on grids, stained with uranyl acetate, and finally subjected to TEM observation. The impact of various treatments on bacterial disruption was assessed using hemolysin sensitivity assays, alkaline phosphatase (AKP) kits, and o-nitrophenyl-β-D-galactopyranoside (ONPG) hydrolysis tests. Initially, bacteria within biofilms underwent different treatments. The culture medium was then collected, and the supernatant was analyzed using the AKP detection kit. Absorbance at 520 nm (OD520) was measured with a microplate reader. Subsequently, biofilms were harvested via sonication and standardized to an optical density (OD600) of 0.1 at 600 nm. Finally, absorbance was measured at 600 nm and 420 nm using a microplate reader. Each group was tested in quintuplicate.

Furthermore, bacterial suspensions were collected, and the well walls and sample surfaces were rinsed with 0.85% NaCl solution. The samples, along with all bacterial suspensions, were transferred to 50 mL centrifuge tubes and vigorously shaken for 5 min. The samples were then removed, and the remaining liquid was centrifuged at 6000 rpm for 10 min. The supernatant was discarded, and the pellets were frozen with liquid nitrogen and temporarily stored in a -80 °C freezer. Subsequently, the samples were placed on dry ice and sent to NovaGenomics (Beijing) Co., Ltd. for analysis using the Illumina NovaSeq 6000. Gene function analysis was conducted using Gene Ontology and the Kyoto Encyclopedia of Genes and Genomes. Differential gene expression analysis was performed using edgeR with genes meeting the criteria of P < 0.05 and log_2_FC > 1 considered differentially expressed.

***Cell culture:*** Bone marrow-derived macrophages (BMDMs), bone marrow-derived dendritic cells (BMDCs), and neutrophils were isolated from C57BL/6J mice and cultured under standard conditions at 37 °C in a humidified atmosphere containing 5% CO₂. Unless otherwise specified, all cells were maintained in RPMI-1640 or DMEM medium supplemented with 10% fetal bovine serum (FBS) and 1% penicillin/streptomycin. Rat bone marrow mesenchymal stem cells (rBMSCs) were obtained from the bone marrow of Sprague–Dawley rats and cultured in DMEM/F12 containing 10% FBS and 1% penicillin/streptomycin at 37 °C under 5% CO₂. The medium was refreshed every 2–3 days, and adherent spindle-shaped cells were expanded for further use.

***In vitro cytocompatibility:*** After co-culturing cells (rBMSCs) with different systems for 24 h, the cells were incubated for 15 min using a Calcein AM/PI cell viability/cytotoxicity assay kit (Sigma). Subsequently, the cells were observed under a fluorescence microscope. To assess the distribution of cells within the specified materials, images of the cytoskeleton staining were captured using confocal microscopy after 24 h of co-culture.

***Validation of Golgi targeting and pH regulation:*** BMDCs were extracted and induced following the steps of previous studies. Cells were inoculated into 96-well cell culture plates with 5 × 10^3^ cells per well and cultured for 24 h. The cells were then exposed to DMEM containing FITC-labelled nanoparticles for 1 h, respectively. After uptake, the culture medium containing the NPs was collected, and the cells were gently washed twice with pre-warmed DMEM culture medium (37 °C) and then incubated in fresh cell culture medium for 2 or 12 h for exocytosis. After exocytosis, the supernatant was collected, rinsed three times with PBS, fixed with 4% paraformaldehyde for 10 min, and stained with 2-(4-amidinophenyl)-6-indolylamidine dihydrochloride (DAPI; Thermo Fisher Scientific) for 10 min. The cells were then subjected to CLSM. Cells cultured on 35 mm glass-bottom dishes were incubated with nanoparticles at 37 °C in standard cell culture medium for 30 min. Subsequently, the culture medium was removed, and the cells were washed with an appropriate volume of buffer solution (Hanks' Balanced Salt Solution with Ca^2+^ & Mg^2+^). After removing the wash solution, a prepared Golgi-Tracker Red staining working solution was added, and the cells were co-incubated at 4 °C for 30 min. The Golgi-Tracker Red staining solution was then recovered, and the cells were washed approximately three times with ice-cold pre-chilled culture medium, followed by incubation in fresh medium at 37 °C for 30 min. A final wash with fresh culture medium was performed, and the cells were typically observed using fluorescence microscopy or laser confocal microscopy. At this stage, the Golgi apparatus exhibited bright, intense fluorescence staining, while other intracellular membrane systems displayed relatively weak fluorescence staining. To assess the regulatory capacity of CS-BT@MZ on Golgi pH under ultrasound, we employed a Golgi pH probe, CPH (2-{4-(sulfamoyl-phenylamino)-7-(1-ethyl-3,3-dimethyl(indolin-2-ylidene)}-3,5-(propane-1,3-diyl)-1,3,5-heptatrien-1-yl)-1-ethyl-3,3-dimethyl-3H-indolium), which exhibits increased fluorescence intensity with rising Golgi pH ^[2]^. In brief, cells cultured on 35 mm glass-bottom dishes were incubated with nanoparticles or Monensin—a Golgi oxidative stress inducer that neutralizes Golgi pH and reduces Golgi protein activity—in standard cell culture medium at 37 °C for 6 h ^[3]^. Subsequently, the cells were incubated with the CPH probe for 25 min prior to CLSM and flow cytometry. Additionally, the expression levels of genes associated with STING activation were assessed using PCR following various treatments, and the activation status of the STING protein was evaluated through immunofluorescence analysis.

***In vitro DC maturation and function assay:*** To prepare BCMs for culturing DC, the supernatants from MRSA biofilms of various treatment groups were collected, centrifuged at 12,000 g for 10 min, and filtered using a 0.22 μm filter (Merck, Germany). The filtered biofilm supernatant was then mixed with DMEM medium (Gibco, USA) at a 1:2 ratio, supplemented with 10% fetal bovine serum (FBS, Gibco, USA) and 1% penicillin/streptomycin (Gibco, USA). It is noteworthy that DCs cultured in BCMs prepared with untreated biofilm supernatant served as the control group. To conduct flow cytometry analysis, distinct subsets of DCs were isolated and incubated for 30 min with fluorochrome-conjugated flow cytometry antibodies, including CD45 (N418), CD80 (16-10-A1), and CD86 (clone GL-1). Following antibody recovery and cell washing, samples were analyzed using a flow cytometer, and the resulting data were processed with FlowJo V10 software. Total RNA was extracted using the TRIzol method according to the aforementioned cell processing protocol. Gene expression was normalized to the housekeeping gene β-actin. Primer sequences used in this study are provided in the supplementary materials (**Table S1**). Additionally, enzyme-linked immunosorbent assay (ELISA) kits were employed to quantify the levels of IFN-β and CXCL10 in the culture supernatants.

***Preparation of CS-BT@MZ@NEs:*** Neutrophils used for nanoparticle loading were isolated from the bone marrow of C57BL/6J mice and administered into recipient mice of the same strain, thereby excluding donor–recipient incompatibility in the present study. Specific pathogen-free C57BL/6J mice (female, 6–8 weeks old, 18–22 g) were maintained under standard specific pathogen-free conditions with free access to food and water. Bone samples were collected from euthanized mice (n = 5) on a sterile workbench. The bone marrow was flushed with PBS and centrifuged at 250g for 4 min, then resuspended in 1ml PBS. A density gradient centrifugation solution was prepared by carefully layering 2ml of 75%, 65%, and 55% (vol/vol) Percoll (Pharmacia) solutions sequentially, with 1ml of the single-cell suspension added on top. Neutrophils were isolated at the interface between the 65% and 75% fractions following centrifugation at 700g for 25 min. The cells were washed three times with ice-cold PBS to remove residual Percoll. The isolated neutrophils were cultured at 37 °C in RPMI 1640 medium containing 1% penicillin/streptomycin in a humidified atmosphere with 5% CO_2_. Cells were stained with FITC anti-mouse Ly-6G/Ly-6C (Gr1) and PE anti-mouse MAIR-IV (CLM-5) antibodies (diluted 1:200 in PBS). Cell purity was assessed by flow cytometry using NovoExpress software and analyzed with FlowJo software. Typically, bone marrow-tendentious neutrophils (BMTNs) can be prepared after 6 h of *in vitro* culture.

CS-BT@MZ@NEs were obtained by incubating neutrophils with CS-BT@MZ. Briefly, neutrophils were cultured in a sterile tube with serum-free medium for 1 h, followed by the addition of 1 mg/ml CS-BT@MZ, and incubated at 37 °C for 1 h. To quantify the amount of CS-BT@MZ in CS-BT@MZ@NEs, FITC-labeled CS-BT@MZ was co-incubated with neutrophils for 1 h following the aforementioned procedure and then analyzed using flow cytometry. To evaluate the migratory capacity of CS-BT@MZ@NEs towards CXCL12 (SDF-1α), a Transwell assay with 3 μm pore polyester membrane inserts was employed. A concentration of 10^6^ per ml DiD-labeled neutrophils or CS-BT@MZ@NEs was seeded into the upper chamber, while CXCL12 (20 ng/mL) was introduced into the lower chamber. After 30 min, fluorescence images of the lower chamber were captured using an inverted fluorescence microscope and subsequently analyzed with ImageJ software. For wells treated with AMD3100 (CXCR4 receptor antagonist), neutrophils were pre-incubated with 1 mg/mL AMD3100 for 15 min prior to following the aforementioned procedure.The biodistribution study commenced with the labeling of nanoparticles using DiR (1,1′-dioctadecyl-3,3,3′,3′-tetramethylindotricarb ocyanine iodide). Subsequently, DiR-NPs and DiR-NPs@NEs were intravenously administered into mice. After 12 h, in vivo imaging was conducted, followed by euthanasia of the mice to harvest major organs (heart, liver, spleen, lungs, kidneys, and bone) for *ex vivo* imaging.

***Establish an "in situ" implant infection model:*** To evaluate the direct therapeutic efficacy of nanorobots, we employed a previously reported method with modifications to establish an "in situ" implant infection model. Sixty male C57 mice (6 weeks old) were randomly divided into six groups: Control, NEs, CS-BT@NEs, CS-MZ@NEs, CS-BT@MZ@NEs, and Van groups. Following anesthesia induction, the left leg of each mouse was shaved and disinfected, the knee joint was exposed through layered incisions, and a bone channel was created on the tibial plateau using a medical drill. Into this channel, 50 μL of 10^7^ CFU/mL *MRSA* was injected, and the wound was meticulously sutured in layers. The mice were then carefully monitored for one day to observe infection progression. Treatment commenced 24 h post-infection, with mice receiving intravenous injections of PBS (Ctrl), CS-BT@NEs, CS-MZ@NEs, CS-BT@MZ@NEs, or Van (10 mg/kg). After 24 h, the mice were subjected to ultrasound (40 kHz, 1.5 W/cm², 1 min per cycle, 5 cycles). To assess the efficacy of different treatments, general observations, clinical imaging evaluations, and bacterial viability tests were conducted at predetermined time points. The knee joint area was recorded using calipers and quantified with the formula: knee joint area = π × a × b (where a and b are the lengths of the semi-major and semi-minor axes, respectively). On postoperative day 20, the implants were extracted, stained, and bacterial detection was performed via CLSM and SEM. The extracted femurs were fixed in EDTA for decalcification over two weeks, followed by preparation of pathological sections for HE and Giemsa staining. Immunofluorescence staining was also conducted for MPO, iNOS, and CD206.

The tibial ends were incised, and bone marrow aspirates were collected using a syringe. The harvested bone marrow suspension was digested in dissociation buffer (100 µg/mL DNase I and 1 mg/mL Collagenase IV) at 37 °C for 30 min. Subsequently, the cell suspension was triturated through a 70 µm filter to obtain a single-cell suspension. Red blood cells were lysed using ACK lysis buffer (Gibco) and washed three times with ice-cold PBS. For immunophenotyping, the cell suspension was incubated with CD16/32 for 10 min and then stained with antibodies against CD45 (S18009D), CD3 (17A2), CD4 (RM4-5), CD8a (53-6.7), CD11b (M1/70), CD11c (N418), CD80 (16-10-A1), CD86 (GL-1), Gr-1 (RB6-8C5), NK1.1 (BM8), CD19 (6DS), and CD138 (281-2). Cytokines (CXCL10, IL10, IL12, and IFN-β) and antibodies (IgM and IgG) in the bone marrow were quantified using ELISA kits as per the manufacturer's instructions. Furthermore, mice with biofilms were administered PBS and CS-BT@MZ@NE. At the conclusion of the treatment, infected tissues were collected and subjected to high-throughput sequencing at Majorbio Bio-Pharm Technology Co., Ltd. The resulting data were analyzed using an interactive analysis platform and differentially expressed genes (DEGs) with an adjusted P-value of less than 0.05 and [log2FoldChange] greater than 1 were selected for enrichment analysis.

***In Vivo Monitoring of Systemic Immune Responses:*** To assess the systemic antimicrobial immune response, we established a contralateral mouse endophyte infection model as follows. First, we established an “in situ” endophyte infection model in the left knee and treated it with the different methods described above.14 Days later, an endophyte infection model was established in the right femur using the same methods described above. The procedure for isolating and staining spleen-associated immune cells was similar to that for bone marrow. Briefly, spleens were dissected and cut into small pieces, enzymatically digested, and mechanically disrupted with a 70 μm cell filter. After removal of erythrocytes with ACK lysis buffer, cell suspensions were co-incubated with CD16/32 for 10 min and then stained on ice with antibodies against CD45 (S18009D), CD3 (17A2), CD4 (RM4-5), CD8a (53-6.7), NK1.1 (BM8), CD11b (M1/70), CD11c (N418), and Gr-1 (RB6-8C5) according to the manufacturer's instructions for staining. Serum cytokines (CXCL1010 and IFN-β) and antibody levels (IgG and IgM) were measured with Elisa kits according to the manufacturer's instructions.

***Prophylactic Vaccine Study:*** To ascertain whether CS-BT@MZ@NEs nanorobots can elicit a long-term immunological memory response, we performed explant retrieval and thorough debridement 14 days post-establishment of an “in situ” implant infection model. Postoperatively, vancomycin was administered intravenously at a dosage of 15 mg/kg daily, fully replicating the clinical treatment of implant infections. General photographs, knee joint area measurements, and magnetic resonance imaging data were collected at predetermined postoperative intervals. Mice were euthanized 21 days after the re-challenge surgery. In vivo bacterial viability assays and Giemsa staining of infected bone were conducted using the aforementioned methods. Spleens were harvested and processed into single-cell suspensions as previously described. The splenic single-cell suspensions were then stained with antibodies against CD45 (S18009D), CD3 (17A2), CD4 (RM4-5), CD8a (53-6.7), CD44 (IM7), and CD62L (MEL-14). Serum and bone marrow IgG antibody levels were assessed as per the established protocol.

***Statistical Analysis:*** Numeric data are presented as mean ± standard deviation unless stated otherwise. For the comparison of two groups, a two-tailed Student's t-test was employed unless indicated otherwise. Multiple group comparisons were performed using one-way analysis of variance (ANOVA) followed by Tukey's post hoc test unless specified otherwise. All calculations and statistical analyses were conducted using Excel 2016 and GraphPad Prism 9 unless noted otherwise. A p-value of less than 0.05 was deemed statistically significant.


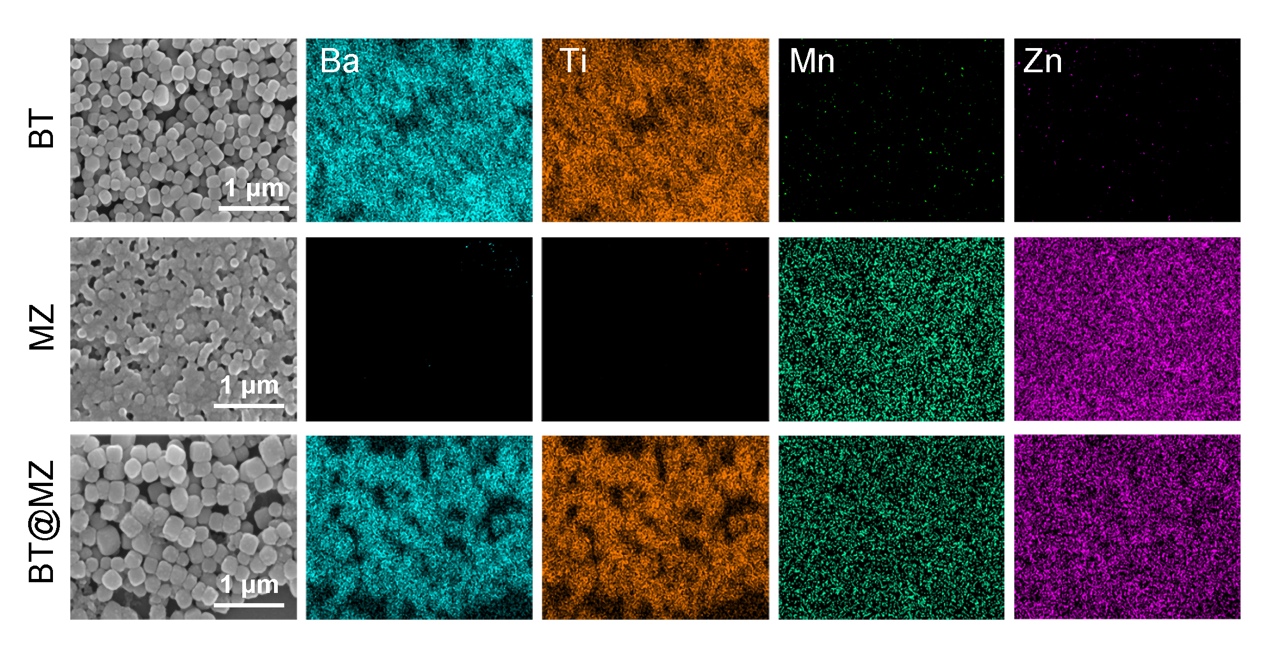


**Figure S1.** SEM images of various samples.


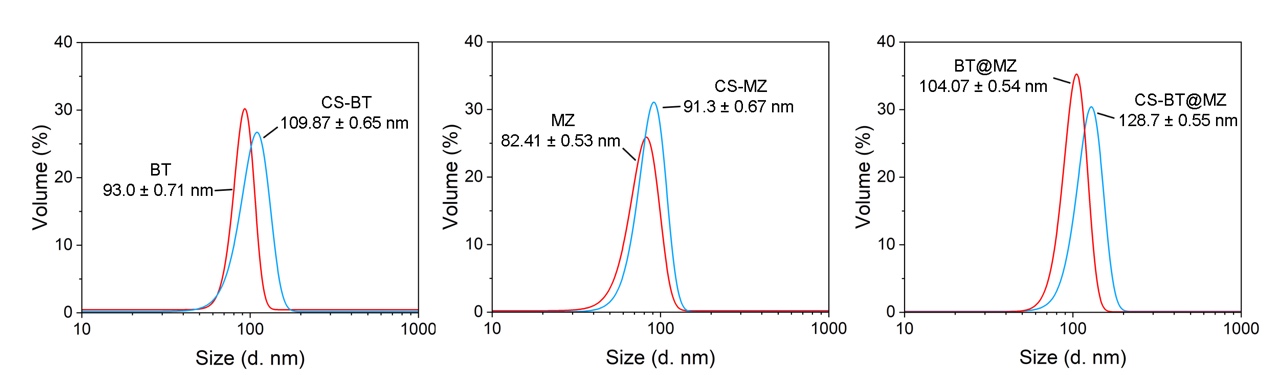


**Figure S2.** DLS of various samples.


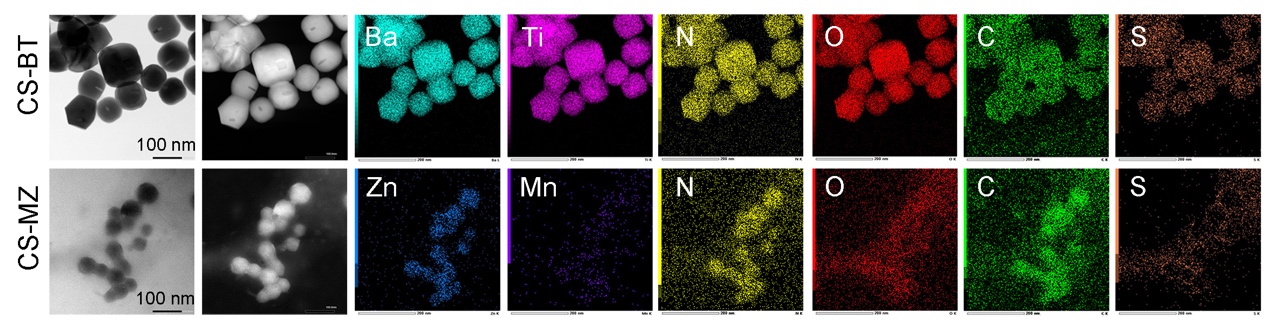


**Figure S3.** TEM images of various samples.


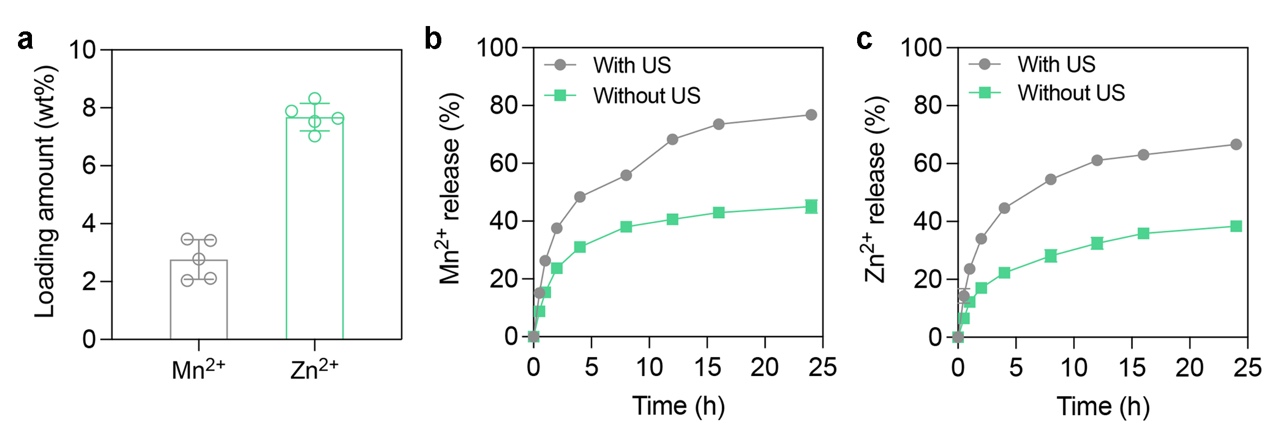


**Figure S4.** (**a**) Loading amount of Mn^2+^ and Zn^2+^ in CS-BT@MZ. The cumulative release profiles of Mn^2+^ (**b**) and Zn2^+^ (**c**) from CS-BTAMZ incubated under varying conditions (With US or Without US). Data are presented as mean ± SD (n = 5 per group), with “n” denoting biologically independent experiments.


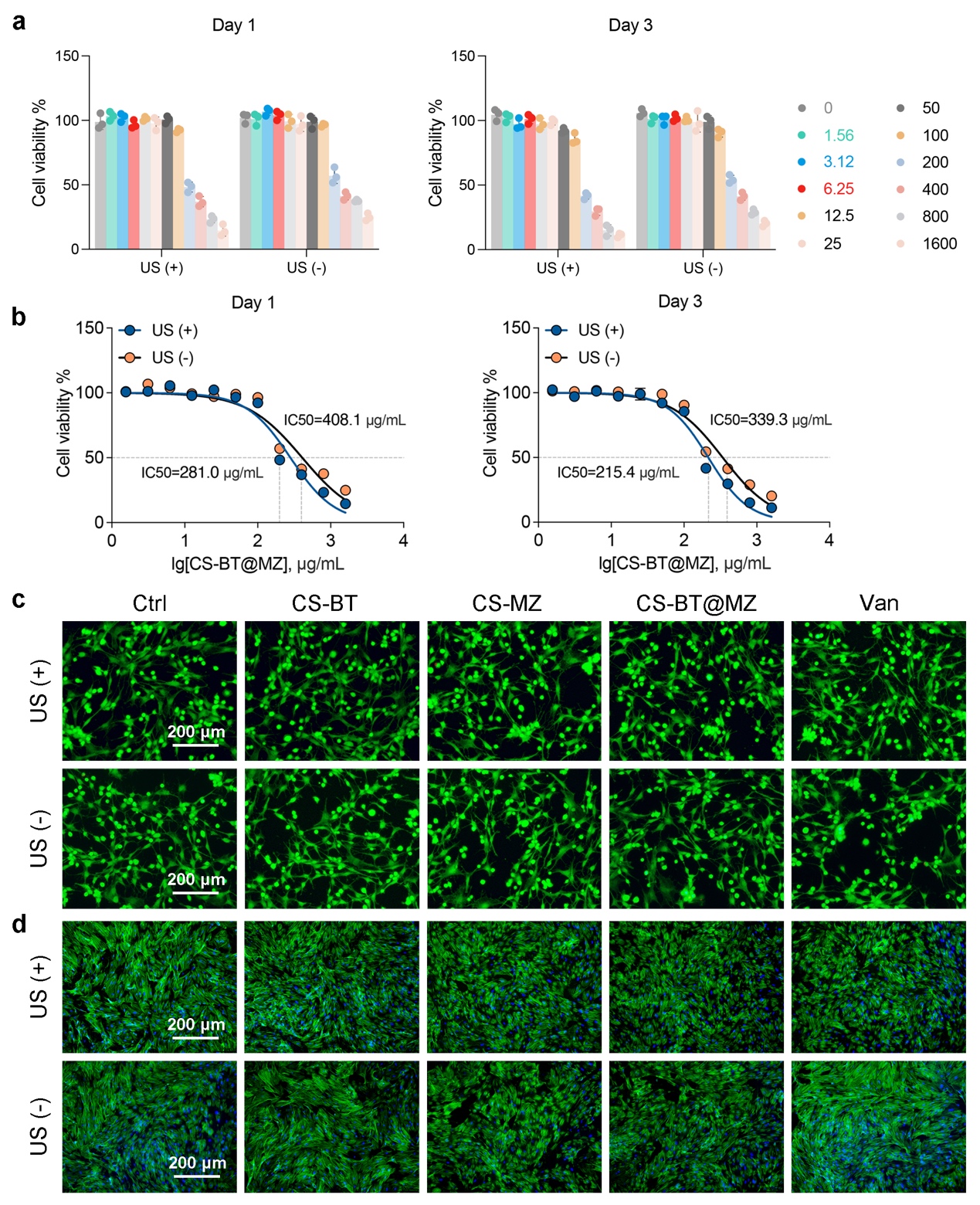


**Figure S5.** ***In vitro* cytocompatibility of various samples.**

(**a**) Cell viability of rBMSCs treated with different concentrations of CS-BT@MZ under US (+) or US (−) conditions for 1 and 3 days. (**b**) Dose–response curves and corresponding IC50 values of CS-BT@MZ toward rBMSCs under US (+) or US (−) conditions on days 1 and 3. (**c**) Live/dead staining images of rBMSCs after treatment with different samples under US (+) or US (−) conditions. (**d**) Fluorescence images of rBMSCs showing cell morphology after treatment with different samples under US (+) or US (−) conditions. Scale bars: 200 μm. Data are presented as mean ± SD (n = 5 per group), with “n” denoting biologically independent experiments.


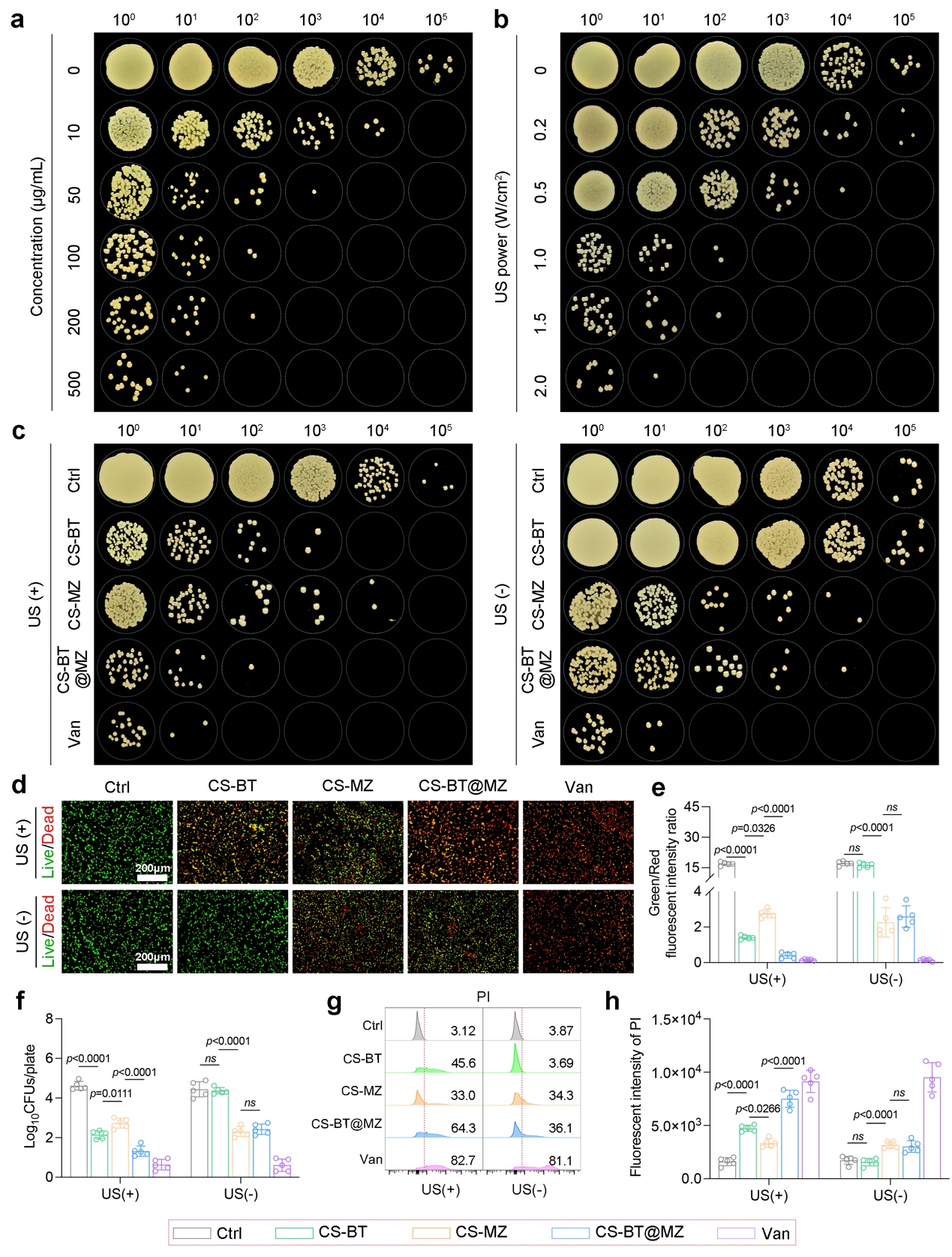


**Figure S6. Anti-bacterial activity of CS-BT@MZ + US.**

(**a**) Antibacterial results of different concentrations of CS-BT@MZ. (**b**) Antibacterial results of CS-BT@MZ at different US power. (**c**) Antibacterial results after different treatments. (**d**) Live/dead staining of bacteria from biofilm (live bacteria, green; dead bacteria, red). (**e**) Quantification of bacteria in biofilms using different systems and vancomycin. (**f**) Quantitative analysis of bacteria in biofilms. (**g**) FCM results of PI staining on extracted biofilm. (**h**) Detection of membrane damage in bacteria through monitoring PI influx. Data are presented as mean ± SD (n = 5 per group), with “n” denoting biologically independent experiments.


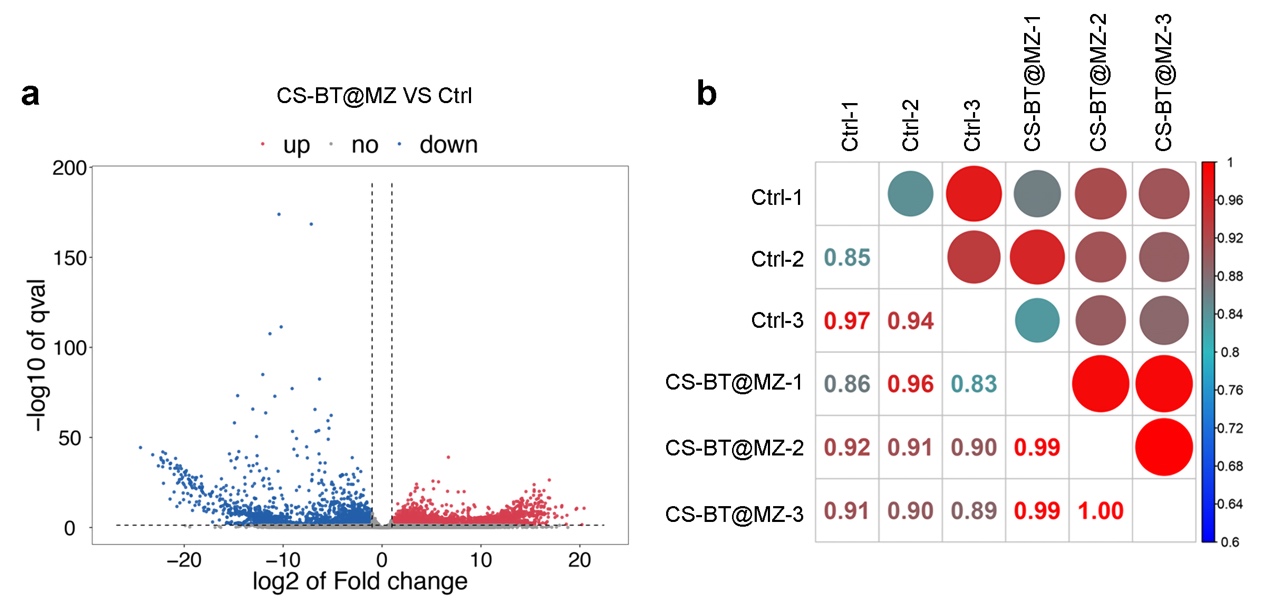


**Figure S7.** (**a**) Volcano plots illustrating diﬀerentially expressed genes. (**b**) PCA analysis of Ctrl and CS-BT@MZ groups.


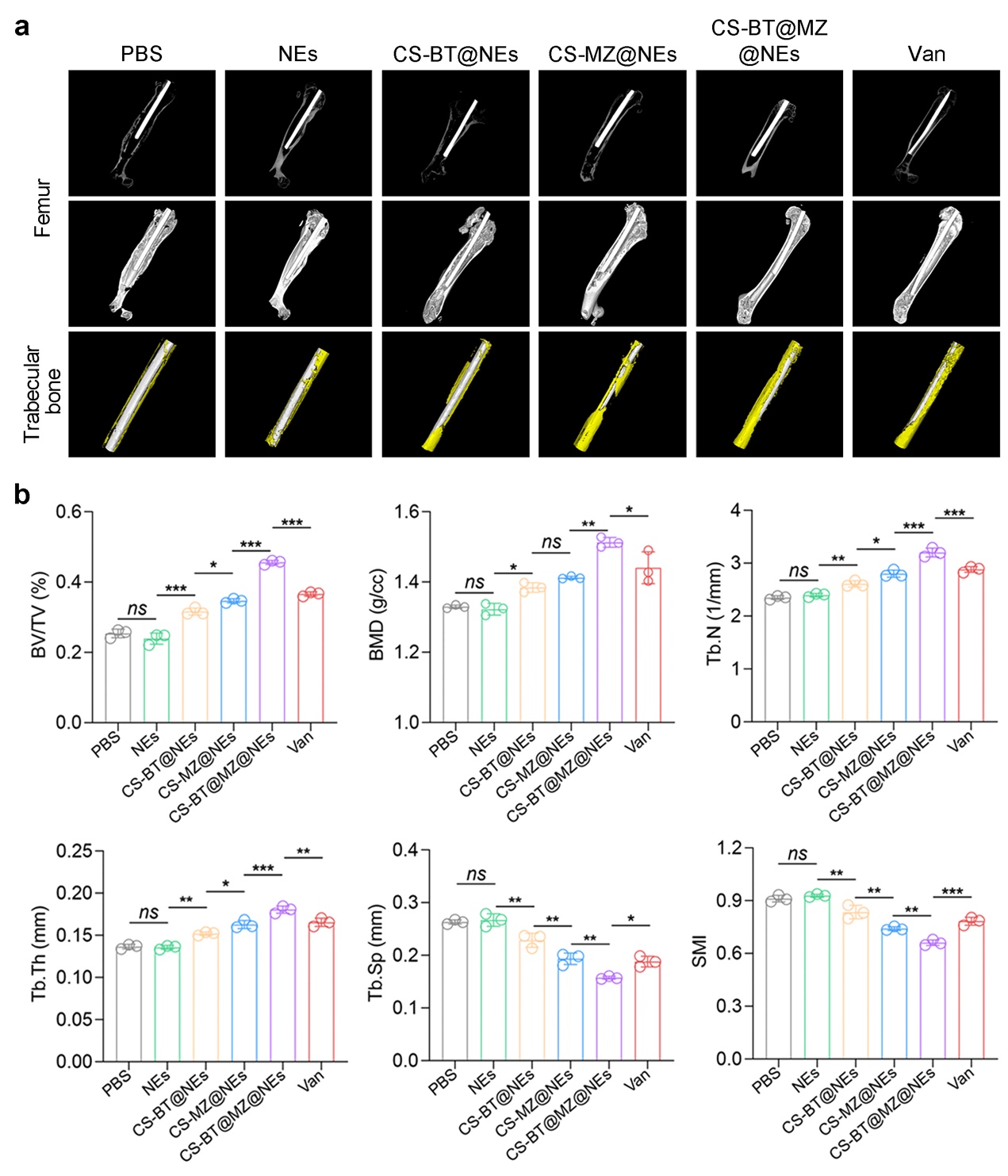


**Figure S8.** **Micro-CT results of the infected bone.**

(**a**) Micro-CT imaging of the infected bone. (**b**) Quantitative analysis results from micro-CT evaluation. Data are presented as mean ± SD (n = 3 per group), with “n” denoting biologically independent experiments.


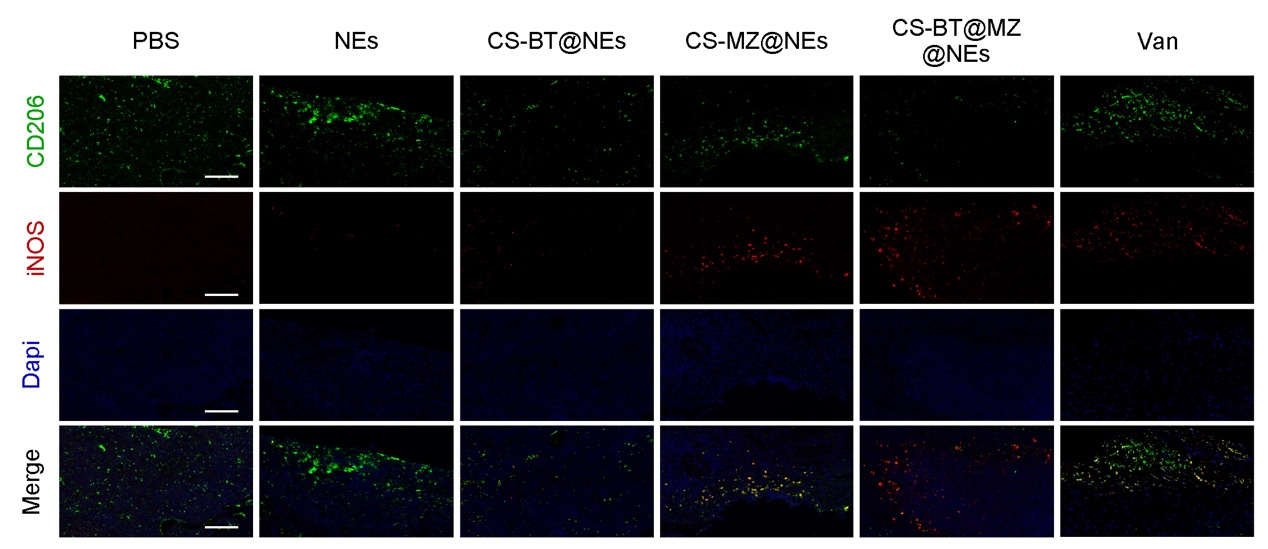


**Figure S9.** Immunofluorescence staining for iNOS and CD206 was performed on infected bone sections after various treatment. Scale bar: 250 μm.


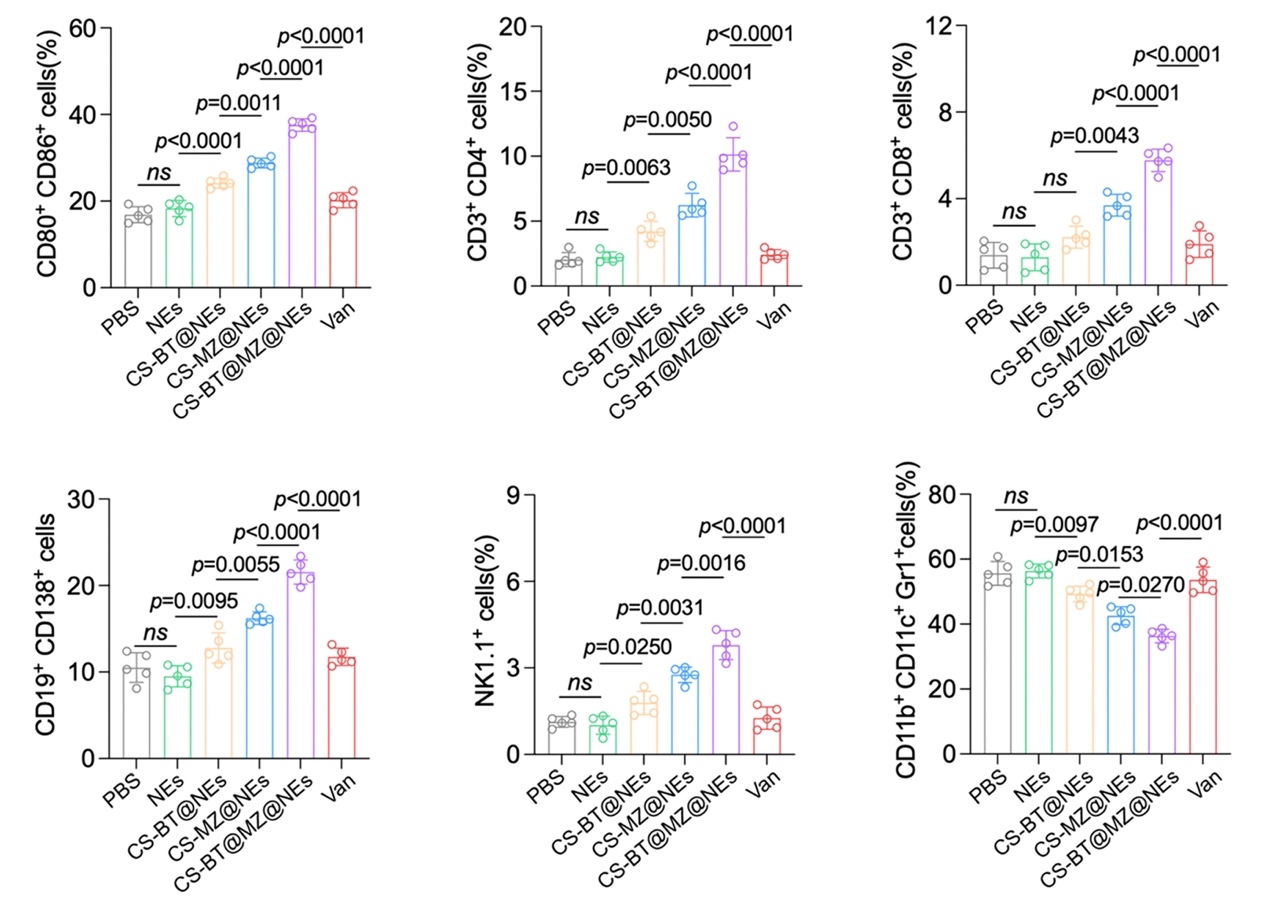


**Figure S10.** Quantification of mature DCs, CD4^+^ T cells, CD8^+^ T cells, plasmablasts, NK cells, and MDSCs cells in the bone marrow on day 21 after treatments. Data are presented as mean ± SD (n = 5 per group), with “n” denoting biologically independent experiments.


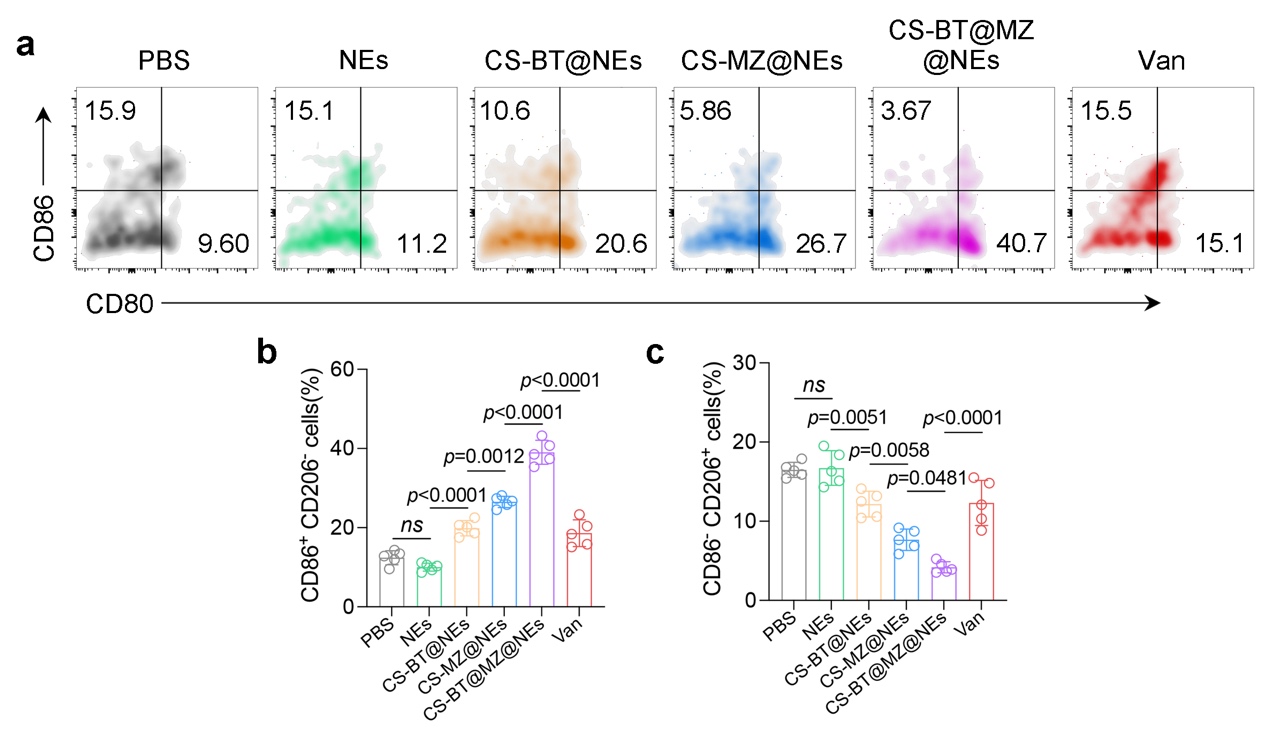


**Figure S11.** Representative flow cytometry plots (**a**) and quantification of M1 macrophages (CD86^+^ CD206^-^) (**b**) and M2 macrophages (CD86^-^ CD206^+^) (**c**) in the bone marrow on day 21 after treatments. Data are presented as mean ± SD (n = 5 per group), with “n” denoting biologically independent experiments.


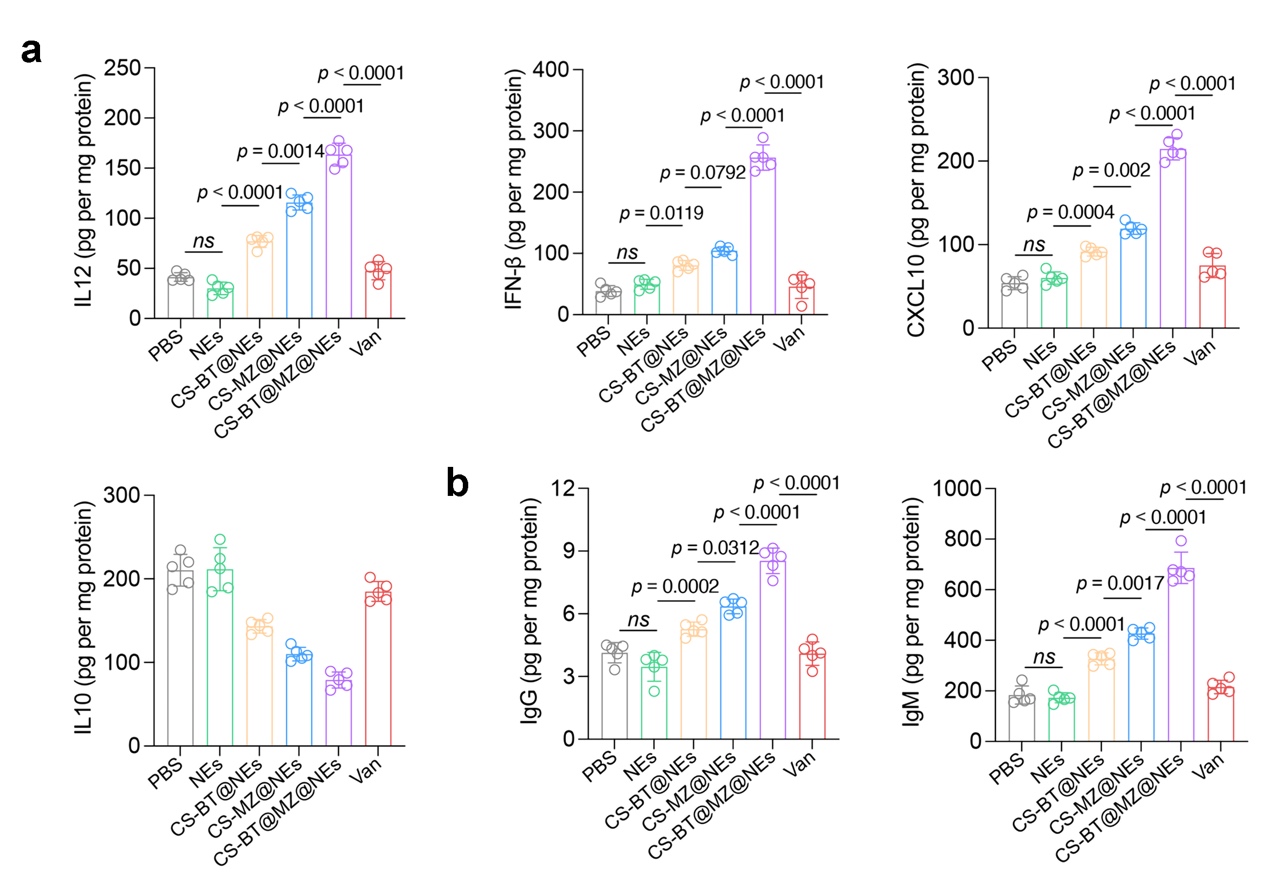


**Figure S12.** (**a**) IL12, IFN-β, CXCL10, and IL10 concentrations in the bone marrow. (**b**) IgG and IgM concentrations in the bone marrow. Data are presented as mean ± SD (n = 5 per group), with “n” denoting biologically independent experiments.


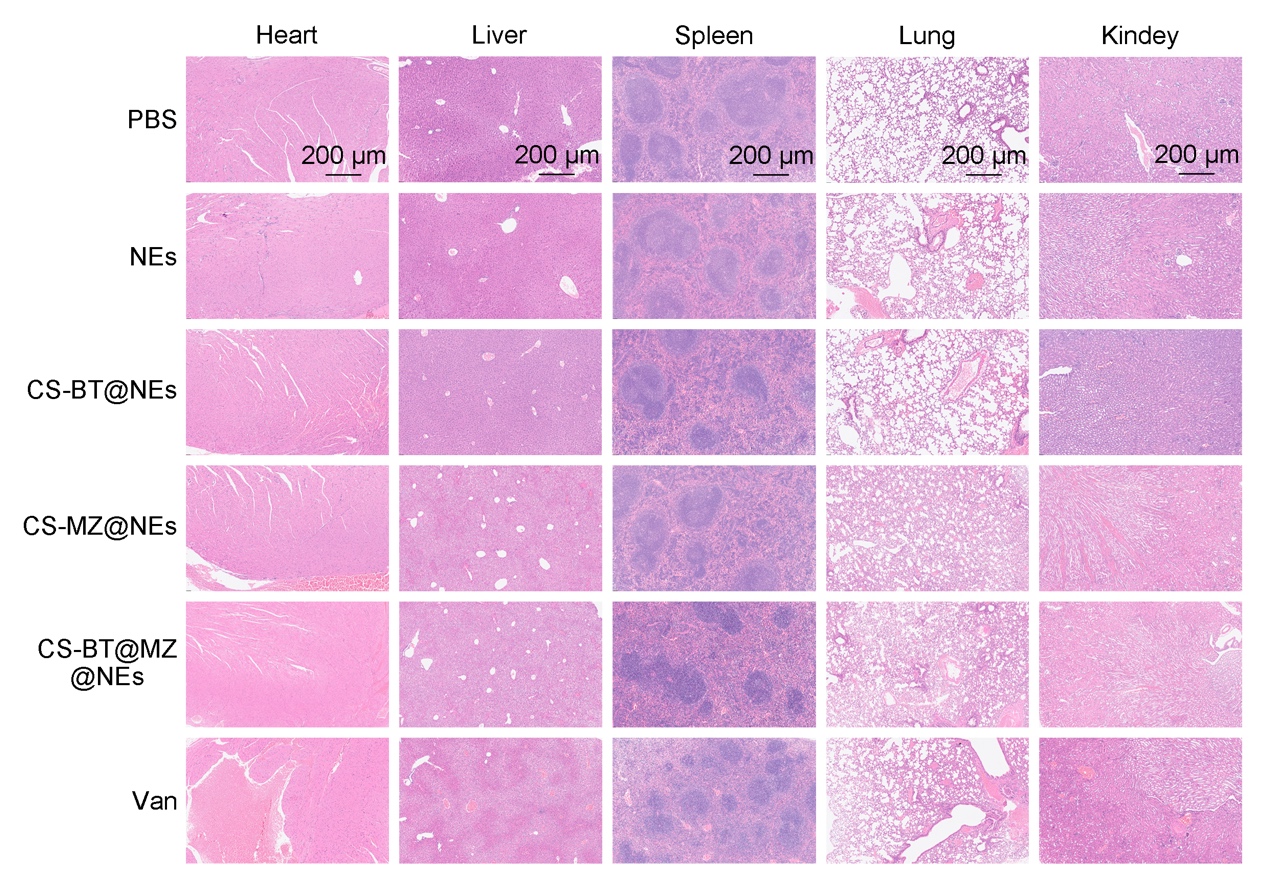


**Figure S13.** H&E staining of heart, liver, spleen, lung, and kidney after different treatments.


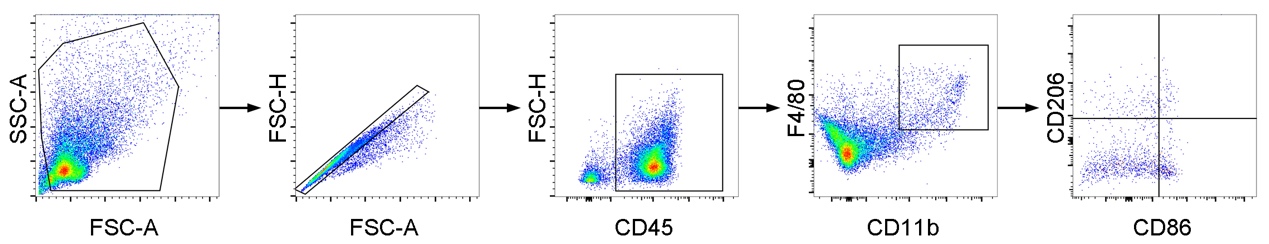


**Figure S14.** Representative gating strategy of macrophages in the bone marrow of the IAIs mice.


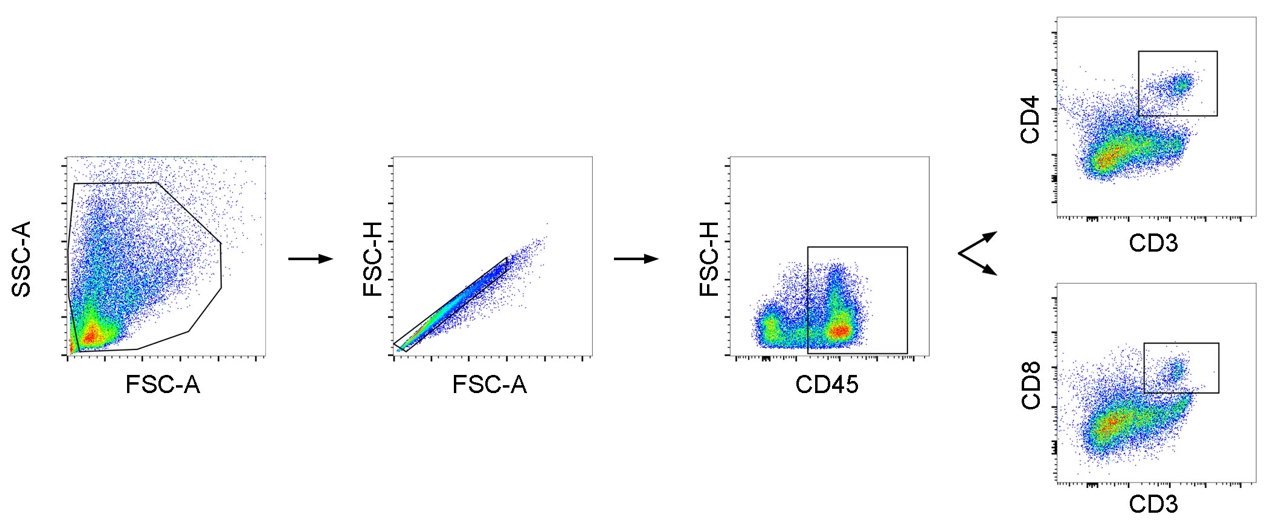


**Figure S15.** Representative gating strategy of CD4^+^ T cells and CD8^+^ T cells.


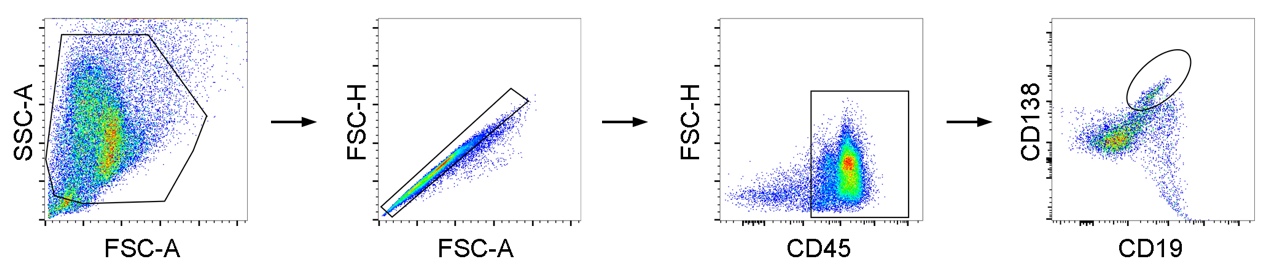


**Figure S16.** Representative gating strategy of plasmablasts cells.


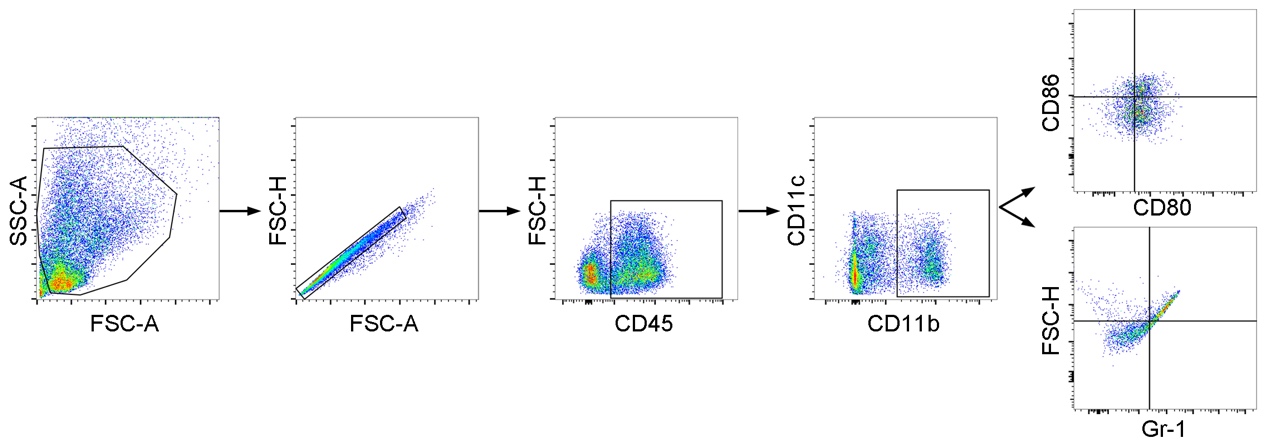


**Figure S17.** Representative gating strategy of DCs and MDSCs cells.


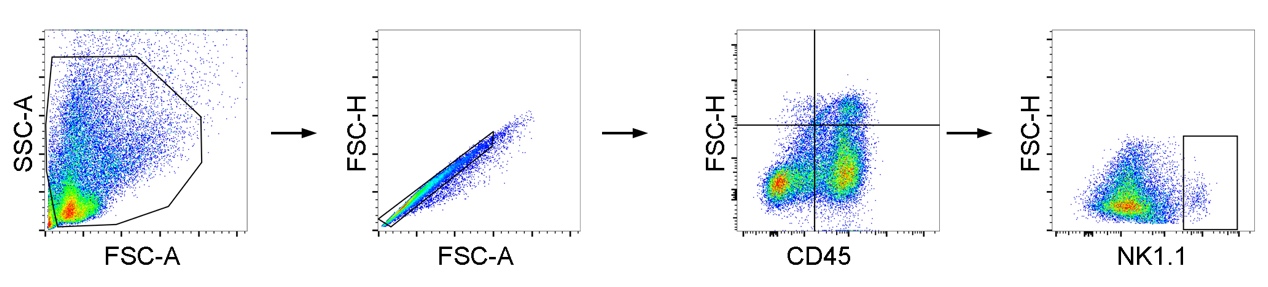


**Figure S18.** Representative gating strategy of NK cells.


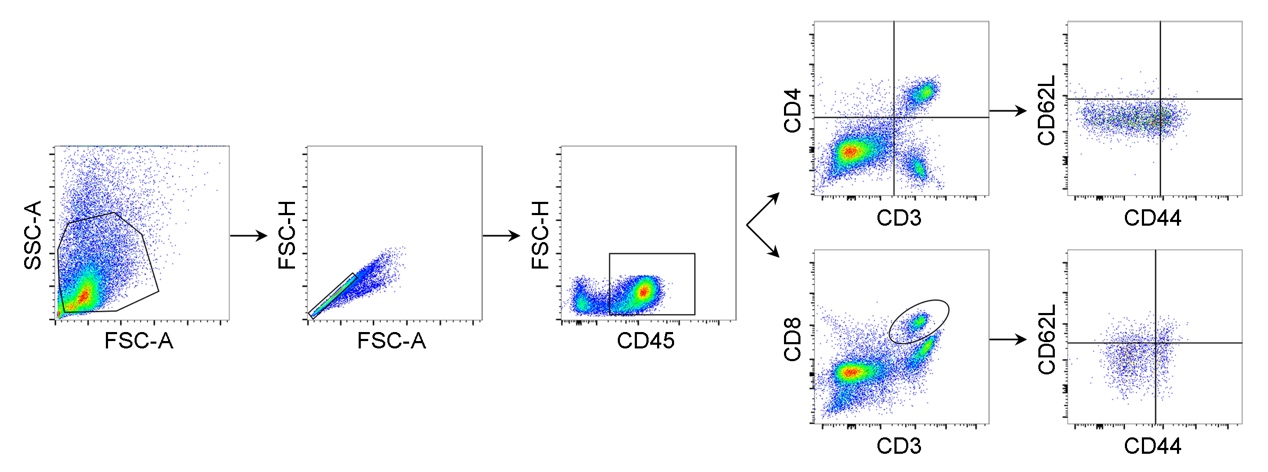


**Figure S19.** Representative gating strategy of CD4^+^ memory cells and CD8^+^ T memory cells.

**Table S1** Primers used in this study.

| Gene and primer direction | Primer sequence (5′to 3′) |
| --- | --- |
| Mouse STING |  |
| Forward | CGGGTTTATTCCAACAGCGTC |
| Reverse | CTGTTTCCGTCTGTGGGTTCT |
| Mouse IRF3 |  |
| Forward | TGGCTGACTTTGGCATCTTCC |
| Reverse | GCTAACCGCAACACTTCTTTCC |
| Mouse TBK1 |  |
| Forward | TCCCTCCCTAAAATACATCCACG |
| Reverse | CACTACCTCCGTCTTCTTGTGG |
| Mouse TNF-α |  |
| Forward | GGGTGATCGGTCCCCAAAGG |
| Reverse | CTCCACTTGGTGGTTTGCTACGA |
| Mouse Ccl3 |  |
| Forward | TTCTGCTGACAAGCTCACCC |
| Reverse | GTCTCTTTGGAGTCAGCGCA |
| Mouse IFNβ |  |
| Forward | GGCGGACTTCAAGATCCCTA |
| Reverse | GAGGACATCTCCCACGTCA |
| Mouse Cxcl10 |  |
| Forward | AAGTGCTGCCGTCATTTTCT |
| Reverse | ACTGGGTAAAGGGGAGTGA |
| Mouse IL-6 |  |
| Forward | TAGTCCTTCCTACCCCAATTTCC |
| Reverse | TTGGTCCTTAGCCACTCCTTC |
| Mouse H-2D |  |
| Forward | GGCTGTCCTGGAACTCACTTTGTAG |
| Reverse | AGCAGTCTCCTCTGGCACCTATG |
| Mouse Timd4 |  |
| Forward | GGCACCACCTCTGACACTTCTTATG |
| Reverse | CCAGACCAAGCCTTCCAAGACATC |
| Mouse Cd40 |  |
| Forward | GCCCTGTTTCTGTCTGTCTGTCTG |
| Reverse | ACTGCCTCTTGGTCTCACTCCTATC |
| Mouse Actin |  |
| Forward | GGCTGTATTCCCCTCCATCG |
| Reverse | CCAGTTGGTAACAATGCCATGT |

**Reference**

[1] J. Yan, G. Wang, L. Xie, H. Tian, J. Li, B. Li, W. Sang, W. Li, Z. Zhang, Y. Dai, *Adv Mater* **2022**, 34, e2105783.

[2] a) R. G. Kurumbail, A. M. Stevens, J. K. Gierse, J. J. McDonald, R. A. Stegeman, J. Y. Pak, D. Gildehaus, J. M. Iyashiro, T. D. Penning, K. Seibert, *Nature* **1996**, 384, 644; b) H. Zhang, J. Fan, J. Wang, B. Dou, F. Zhou, J. Cao, J. Qu, Z. Cao, W. Zhao, X. Peng, *Journal of the American Chemical Society* **2013**, 135, 17469; c) H. Wang, Z. He, Y. Yang, J. Zhang, W. Zhang, W. Zhang, P. Li, B. Tang, *Chemical science* **2019**, 10, 10876; d) H. Wang, Y. Yang, F. Huang, Z. He, P. Li, W. Zhang, W. Zhang, B. Tang, *Analytical Chemistry* **2020**, 92, 3103.

[3] a) L. Chen, P. Jiang, X. Shen, J. Lyu, C. Liu, L. Li, Y. Huang, *Small* **2023**, 19, e2204747; b) A. Eisenberg-Lerner, R. Benyair, N. Hizkiahou, N. Nudel, R. Maor, M. P. Kramer, M. D. Shmueli, I. Zigdon, M. Cherniavsky Lev, A. Ulman, J. Y. Sagiv, M. Dayan, B. Dassa, M. Rosenwald, I. Shachar, J. Li, Y. Wang, N. Dezorella, S. Khan, Z. Porat, E. Shimoni, O. Avinoam, Y. Merbl, *Nat Commun* **2020**, 11, 409.
